# A full lifespan model of vertebrate lens growth

**DOI:** 10.1098/rsos.160695

**Published:** 2017-01-18

**Authors:** Hrvoje Šikić, Yanrong Shi, Snježana Lubura, Steven Bassnett

**Affiliations:** 1 Department of Ophthalmology and Visual Sciences, Washington University, St Louis, MO 63130, USA; 2 Department of Mathematics, University of Zagreb, Zagreb, Croatia

**Keywords:** lens, organ growth, branching process, stochastic, model

## Abstract

The mathematical determinants of vertebrate organ growth have yet to be elucidated fully. Here, we utilized empirical measurements and a dynamic branching process-based model to examine the growth of a simple organ system, the mouse lens, from E14.5 until the end of life. Our stochastic model used difference equations to model immigration and emigration between zones of the lens epithelium and included some deterministic elements, such as cellular footprint area. We found that the epithelial cell cycle was shortened significantly in the embryo, facilitating the rapid growth that marks early lens development. As development progressed, epithelial cell division becomes non-uniform and four zones, each with a characteristic proliferation rate, could be discerned. Adjustment of two model parameters, proliferation rate and rate of change in cellular footprint area, was sufficient to specify all growth trajectories. Modelling suggested that the direction of cellular migration across zonal boundaries was sensitive to footprint area, a phenomenon that may isolate specific cell populations. Model runs consisted of more than 1000 iterations, in each of which the stochastic behaviour of thousands of cells was followed. Nevertheless, sequential runs were almost superimposable. This remarkable degree of precision was attributed, in part, to the presence of non-mitotic flanking regions, which constituted a path by which epithelial cells could escape the growth process. Spatial modelling suggested that clonal clusters of about 50 cells are produced during migration and that transit times lengthen significantly at later stages, findings with implications for the formation of certain types of cataract.

## 1. Introduction

An important and still largely unresolved issue in developmental biology is the nature of the processes that specify the size and shape of organs. A number of experimental systems have been deployed to tackle this challenging question. Studies on wing development in *Drosophila* have been particularly informative, helping identify underlying signalling networks [1] and the potential role of mechanical feedback [2] in the growth process. The pathways that regulate growth of the *Drosophila* imaginal disc are also present in higher organisms, where they presumably play analogous roles. However, modelling organ growth in vertebrates is a daunting prospect because of the size and complexity of the structures involved.

The lens of the vertebrate eye offers an opportunity to model the growth of a simple vertebrate organ across the entire lifespan and, by doing so, identify key mathematical determinants of the growth process. From a modelling standpoint, the lens has several advantages. Its role in image formation requires a smooth ellipsoidal shape. It contains only two cell types: epithelial cells and fibre cells. The lens cell population (10^5^–10^6^ cells) is sizeable, but certainly accessible using modern computing tools. The prismatic fibre cells that make up the majority of the lens volume are packed closely together, leaving little or no space between. Importantly, fibre cells do not turnover; all the fibres that differentiate in the course of development are retained in the body of the lens.

We previously quantified the distribution of proliferating cells on the spherical anterior lens surface [3,4] and used those data to formulate a first generation, branching process model of lens growth [5]. Using an expanded, dynamic version of that model, we here report to our knowledge, the first full lifespan growth model for a simple vertebrate organ system, the ocular lens. We were able to follow lens growth through more than 1000 iterative cycles, during which lens volume increases more than 4000-fold. Remarkably, for an organ whose development appears to depend on a stochastic growth engine, the variance in the process was much smaller than predicted by the cell power law [6]. Furthermore, we found that modest adjustment of just two parameters, the rate of increase in cellular footprint area and the proliferation rate, was sufficient to capture the entire growth behaviour of the lens, including radial increment, zonal organization and patterns of cellular immigration–emigration.

## 2. Methods

### 2.1. Age-dependent growth parameters

#### 2.1.1. S-phase labelling

Mice (C57BL/6 J) were obtained from Jackson Laboratory (Bar Harbor, ME). S-phase cells were identified following incorporation of 5-ethynyl-2'-deoxyuridine (EdU; Invitrogen, Carlsbad, CA, USA), as described [3]. EdU was administered by intraperitoneal injection and mice were killed 1 h later by CO_2_ inhalation. Eyes were fixed in 4% paraformaldehyde/phosphate-buffered saline, embedded in paraffin, and sectioned (4 µm) in the midsagittal plane. EdU-positive cells were visualized using Click-iT (Invitrogen) chemistry with Draq5 (Cell Signaling Technology, Danvers, MA, USA) as a nuclear counterstain. Three sections from each of three lenses were used for each time point.

#### 2.1.2. Measurement of radial growth

Radii of intact, fixed embryonic and early postnatal lenses were determined from digital images. Measurements were supplemented by published data collected from adult mouse lenses [7,8].

#### 2.1.3. Determination of fibre cell dimensions in the equatorial plane

Fibre cell width (*w*) was determined from the spacing of nuclei in the meridional rows that form during the early stages of fibre cell differentiation. These measurements were made on confocal image stacks collected in the course of an earlier study [4]. Fibre cell thickness (*ρ*) was determined from vibratome sections of fixed lenses stained with ActinGreen (Invitrogen) to label the sub-membrane actin cytoskeleton and thus highlight the membrane profiles. Measurements were made on fibre cells located 10 cell layers below the equatorial lens surface.

### 2.2. Development of a dynamic model

#### 2.2.1. Anatomy of the growth process, proliferative zones and the applicability of a stochastic model

**Figure 1.**
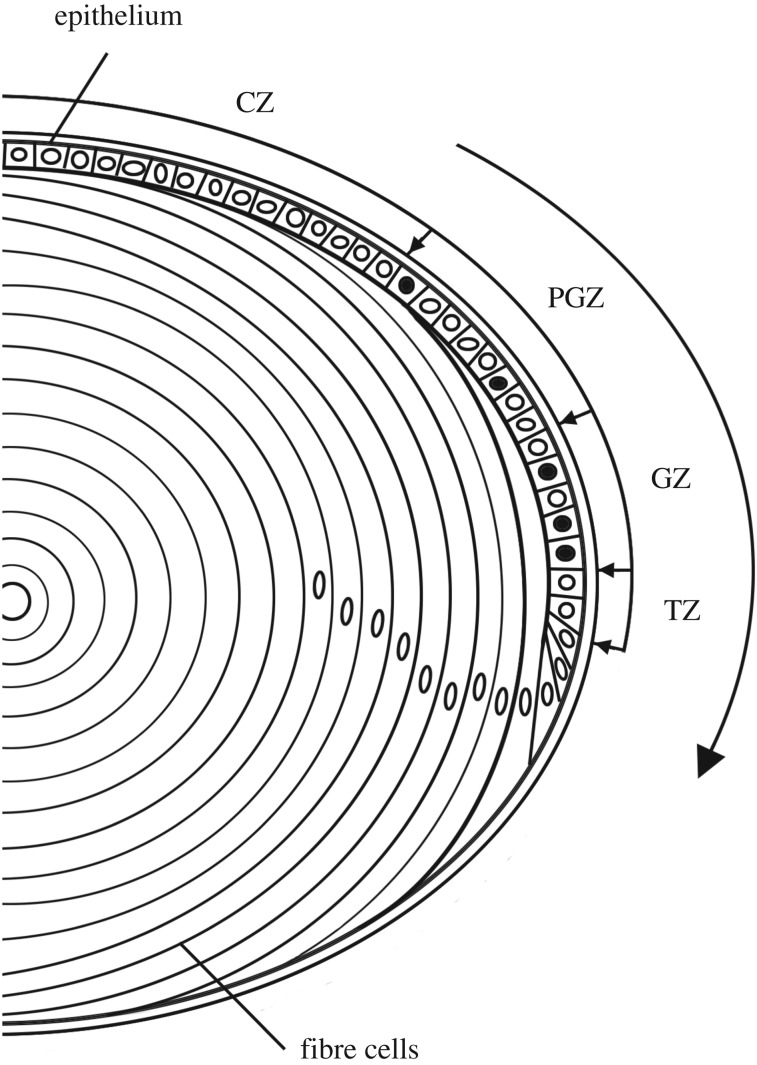
Anatomy of lens growth. The lens is composed of two cell types: epithelial cells and fibre cells. The epithelium can be divided into four zones with respect to regional proliferation rates. S-phase cells (black nuclei) are not observed in the central zone (CZ), but are detected occasionally in the pre-germinative zone (PGZ) and are most numerous in the germinative zone (GZ). At the edge of the epithelium, in the transition zone (TZ), cells exit the cell cycle. Driven by mitotic activity in the PGZ and GZ, epithelial cells are displaced toward the equator in the direction shown by the large arrow. Cell division and differentiation are ongoing, resulting in continuous formation of fibre cells and lifelong macroscopic growth of the lens.

The anatomy of the lens growth process is well understood (figure 1). Mitosis in the anterior epithelium results in displacement of cells from the epithelial margin [9], the ‘penny pusher’ effect [4]. Stimulated by growth factors present in the vitreous humour [10], displaced cells differentiate into fibre cells and are incorporated into the lens body, increasing its volume and surface area. Mitosis is restricted to the epithelium but the rate of cell division varies greatly with latitude. The epithelium can be subdivided into four zones with respect to the local rate of cell proliferation [4]. From the anterior pole to the equator these are: the central zone (CZ), pre-germinative zone (PGZ), germinative zone (GZ) and transition zone (TZ). In adult lenses, the CZ contains mitotically quiescent cells, the PGZ and GZ contain proliferating cells (the rate of cell proliferation is highest in the latter), and the TZ contains cells that have withdrawn from the cell cycle but yet to differentiate. Cells move toward the equator, traversing the various zones before exiting the TZ and differentiating into fibre cells. Of note, apoptotic cells are rarely if ever detected in healthy lenses [4].

The young lens grows quickly, fuelled by rapid cell division in the PGZ and GZ. In the 30 days following its formation on embryonic day 10.5 (E10.5), the mouse lens increases in volume by more than 4000-fold. It transforms from a hollow ball of ≈1200 cells, into a fully formed lens; a solid, ellipsoidal structure containing ≈200 000 cells (≈50 000 epithelial cells and ≈150 000 fibre cells). Initially, all epithelial cells are dividing, but from E14 onwards the proliferation index drops below 100% and probabilistic models are applicable. Here, we modelled lens growth from E14.5 onwards.

#### 2.2.2. Model derivation

We previously developed a branching process-based model to examine how the geometry of the lens constrains its growth [5]. We developed formulae relating proliferation in the various zones to the movement of cells across zonal borders and, ultimately, their deposition as differentiated fibres in the body of the lens. The model was tested over the period from one to three months of age, under the simplifying assumption that age-dependent variables remained unchanged over this relatively short interval. In actuality, almost all parameters vary significantly with age and an adequate full lifespan model must account for this. In this study, therefore, we incorporated newly acquired data on age-dependent changes in fibre cell cross-section, cellular compaction, proliferation rate, cell-cycle period, cellular footprint area (*a*) and the proportional contribution of each zone to the epithelial surface (*η*). Model simulations were benchmarked against data on lens size, cell proliferation rates and published measurements of epithelial population dynamics [4]. Derivation of the static model has been described [5]. The same axioms were employed here in formulating the dynamic full lifespan model (appendix A). Cell dynamics in the proliferative zones are defined by the following conditions:

$$\frac{X_{i}(t+1)a_{i}(t+1)}{\eta_{i}(t+1)}=2\rho (t+1)w(t+1)u+\frac{X_{i}(t)a_{i}(t)}{\eta_{i}(t)},$$

where *X_i_*(*t*) is the number of cells at time *t* in zone *i*, i=1 (CZ),2(PGZ),3(GZ),4(TZ), *u* = the part of the number of cells that move from TZ into the fibre compartment; which consists of elongated cells with hexagonal equatorial plane intersections given by the length *ρ*(*t*) and width *w*(*t*) of the hexagons. These equations lead to a linear system with a unique set of solutions (appendix A), providing us with the number of cells that move between zones.

## 3. Results

### 3.1. Formation of proliferative zones

**Figure 2.**
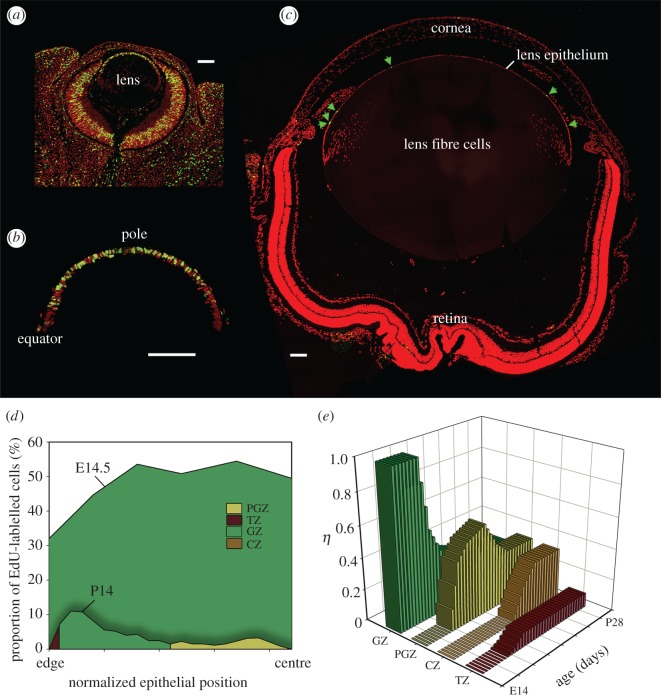
Formation of proliferative zones. S-phase cells (green) were visualized in sections of developing mouse eye. At E14.5 (*a*,*b*), S-phase cells are distributed uniformly but, by P14 (*c*), they are most numerous in the peripheral epithelium (green arrows). Labelling index profiles were computed (*d*). For the examples shown (E14.5 and P14), the proliferation rate is higher throughout the epithelium at the earlier stage. By P14, three zones (TZ, GZ and PGZ) are discernible, the fourth (CZ) appears a few days later. The fractional contribution (*η*) of each zone to the surface area varies initially but, by P28, values have stabilized (*e*). Scale bar (*a–c*) = 100 µm.

The fraction of the anterior lens surface covered by each zone (*η*) was determined previously: *η*_CZ_ = 0.32, *η*_PGZ_ = 0.33, *η*_GZ_ = 0.26, *η*_TZ_ = 0.09 [5]. In adult lenses, many epithelial cells are in *G*_0_ [11] but this is not the case in the embryo, where Ki67 labelling (a marker for cycling cells) approaches 100% [12]. S-phase cells are distributed uniformly across the embryonic lens epithelium [12] which, as a result, can be considered to represent a single GZ (i.e. *η*_GZ_ = 1). To delineate the zones in an embryonic lens, the latitudinal distribution of S-phase cells was visualized in lens sections from EdU-treated embryos (figure 2). S-phase cells were numerous throughout the epithelium at E14.5 (figure 2*a,b*) and the labelling index correspondingly high, with values approaching 50% across much of the epithelium (figure 2*d*). By postnatal day 14 (P14), however, the labelling index at all latitudes had declined, and S-phase cells were concentrated in the periphery (figure 2*c*). The labelling index profile (figure 2*d*) indicated that three zones (TZ, GZ and PGZ) were present. The fourth zone, the CZ, formed between P14 and P21 (figure 2*e*).

**Figure 3.**
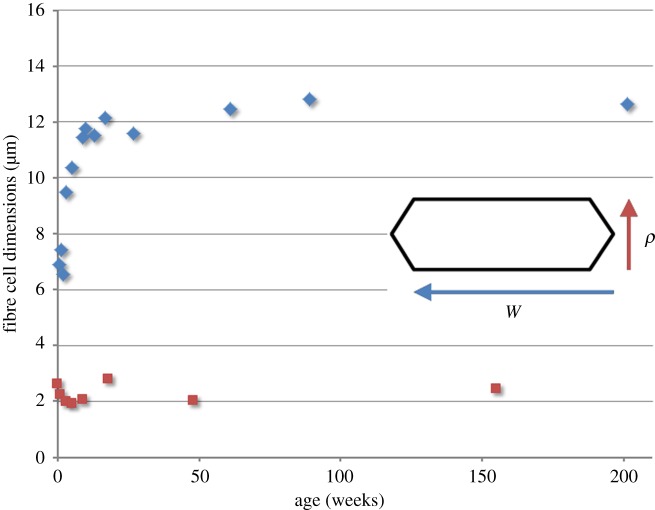
Age-dependent variation in fibre cell cross-sectional shape. In the equatorial plane, fibre cells have a flattened hexagonal profile. The broad faces of the hexagons are oriented parallel to the lens surface. Variations in cross-sectional area (the product of width (*w*, blue diamond) and thickness (*ρ*, orange square)) were incorporated into the lens growth model. Note that fibre cell width increases significantly over the first few months of age, whereas cell thickness is relatively constant across the lifespan.

The cross-sectional area of newly formed fibre cells is an important determinant of radial growth. Previously, we assumed that width and thickness of the prismatic fibre cells were constant over time. To more accurately follow the growth process across the lifespan, we used confocal microscopy to measure the size and shape of fibres in the equatorial plane (figure 3). These experiments revealed that the width (*w*) of the fibres changed significantly during embryonic and early postnatal lens development before stabilizing by ≈6 months of age. By contrast, fibre cell thickness (*ρ*) was relatively constant across the lifespan.

### 3.2. Early growth is characterized by a shortened cell cycle

Cell-cycle duration in adult lens epithelia is 24 h, of which 12 h are required for S-phase [13]. In the embryo, lens growth is so rapid that even if all the epithelial cells were cycling, a 24 h cycle time/12 h S-phase would be insufficient to support the observed increase in cell number. It is not uncommon for the cell cycle to be abbreviated in developing systems. For example, in the cleavage cycles marking the earliest stages of embryogenesis, the *G*_1_ and *G*_2_ phases are skipped, resulting in cycle times measured in minutes rather than hours [14]. Later, the cycle begins to lengthen. In the ventricular epithelium of the cerebral wall, for example, cycles lengthen from 8.1 h at E11 to 18.4 h at E16 [15]. We developed a model relating the proliferation rates of lens cells to their cell-cycle duration (appendix B) and used it to calculate the degree to which the cycle must shorten to be consistent with empirical data. The model suggests cell-cycle durations of 8 h with a 5 h S-phase (8/5 h) from E10–E12, 10/5.5 h from E 12–E14, 12/6 h from E14–E16, 16/8 h from E16–E18 and 24/12 h from E18 onwards.

### 3.3. Population dynamics

#### 3.3.1. Epithelial cells

Unlike other ectodermally derived structures (e.g. epidermis), differentiated lens cells are not shed from the tissue surface. Instead, they are incorporated into the body of the lens, increasing its volume and surface area. Thus, ongoing cell division in the proliferative zones of the epithelium causes the area of the zones to increase (as the surface area of the lens increases). In such a ‘self-inflating’ system, the number of epithelial cells must be regulated tightly if positive feedback loops are to be avoided.

We modelled epithelial cell dynamics using input data on zonal proliferation rates and cellular footprint areas (*a*). Data were collected from E14.5 until the end of the lifespan. Proliferation rates and derived *η* values are presented in table 1 and figure 2*d*,*e*. Corrections for fibre cell compaction were applied from six months, as described below. Simulations were based solely on these restricted input data and a random number generator used to mimic branching mechanisms for individual cells.

**Table 1.** Table of GZ (*r*) and PGZ (*q*) proliferation rates.

	*q*(%)	*r*(%)
E14. cycle 1	0	82
E14. cycle 2	0	73
E15. cycle 1	0	64
E15. cycle 2	0	56
E16–E17. cycle 1	0	48
E16–E17. cycle 2	0	39
E16–E17. cycle 3	0	30
E18	0	25
E19	0	24
E20	0	23
P1	17	30
P2	17	30
P3	17	30
P4	16	30
P5	16	30
P6	16	30
P7	13	27
P8	7	20
P9	7	20
P10	7	20
P11	7	20
P12	7	20
P13	7	19
P14	7	19
P15	7	19
P16	7	19
P17	6	15
P18	6	15
P19	6	15
P20	6	15
P21	6	14
P22	6	14
P23	6	12
P24	5	12
P25	5	10
P26	5	10
P27	5	9
P28	5	9
two to three months	2	6
four to six months	0,5	1,5
seven months to 2 years	0,3	1,2

**Figure 4.**
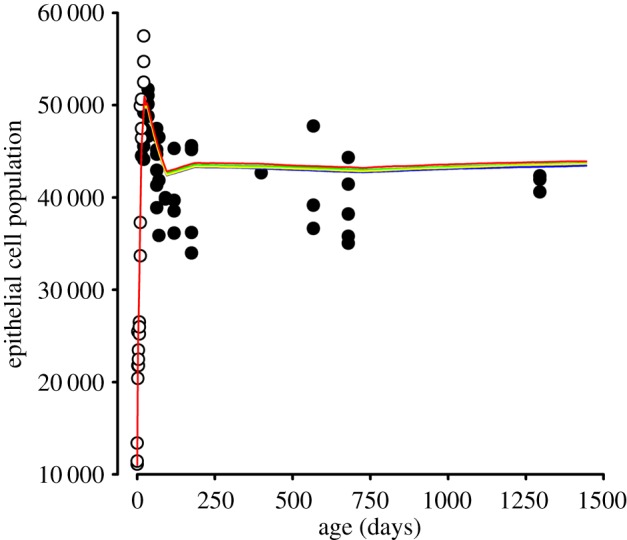
Population dynamics in the mouse lens epithelium from E14.5 (model day 0) until the end of life. Population data were determined directly (open circles) or adapted from published values (filled circles; [4]). Results of five independent model simulations are shown (coloured lines). Note the concordance between model runs.

Modelling suggests that the epithelial cell population rises sharply from E14.5-P14 (figure 4), reaching a maximum at ≈4 weeks (w) of age. The rapid increase is a consequence of shortened cell cycles at embryonic stages (appendix B), a uniformly high proliferation rate (figure 2) and relatively small *a* at all latitudes (which serves to maximize zonal populations). The epithelial cell population overshoots at 4 w, when, for a brief period, it exceeds 50 000. The subsequent population decrease (to ≈43 000 cells by 12 w) reflects a reduction in zonal proliferation rates and an increase in *a* for all zones. Surprisingly, the growth trajectory of the epithelial population depended simply on the relative rates of change in proliferation and footprint area (appendix C). From 12 w onward, the epithelial population remained constant, despite continuing production of cells in the GZ and PGZ and associated macroscopic growth of the lens. Of note, model runs were nearly superimposable, the stochastic nature of the underlying growth mechanism notwithstanding.

Model simulations were compared with experimental determinations of postnatal lens epithelial populations [4] and newly acquired data on embryonic lens epithelia (table 2). Despite some scatter, the empirical data were consistent with model predictions. Both simulations and measurements suggested a transient overshoot in cell population at ≈4 w declining, thereafter, to a smaller but more stable population.

**Table 2.** Epithelial cell populations during early development.

	epithelial cell population		
age	animal 1	animal 2	animal 3
E14.5	13 385	11 092	11 431
E16.5	25 465	20 398	21 788
E18.5	23 468	21 788	22 741
P1	26 494	25 211	25 977
P3	49 911	33 677	37 283
P7	50 627	46 410	47 447
P14	57 483	52 476	54 724

#### 3.3.2. Fibre cells

**Figure 5.**
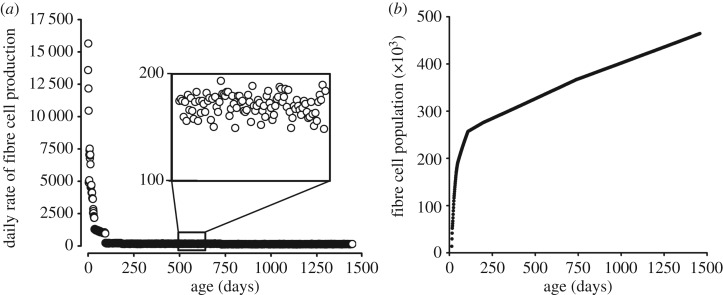
Fibre cell production and accumulation. (*a*) Early in development, fibre cells are produced at a rapid rate (more than 15 000 cells per day at E14.5 (model day 0)). Later, the rate of production falls to 100–200 cells per day (inset). The number of fibres increases throughout life, rapidly in the young lens, more slowly at later time points (*b*). By the end of the lifespan, the lens contains nearly half a million fibre cells.

The lens growth engine (cell division in the proliferative zones) is particularly active during embryonic and early postnatal life leading, at peak, to the deposition of more than 15 000 fibre cells per day (figure 5*a*). However, by three months, modelling indicates that the rate of fibre production declines to a few 100 cells per day (figure 5*a*, inset). The reduction reflects a decline in PGZ and GZ proliferation rates, coupled with an increase in *a* at all latitudes that serves to deplete the proliferative zones of cells. The slow but steady addition of fibres over the life of the mouse (almost 4 years in our laboratory) results in a substantial accumulation of cells. By the end of the lifespan, modelling suggests that the lens contains 400 000–500 000 fibres (figure 5*b*).

### 3.4. Fibre cell compaction

**Figure 6.**
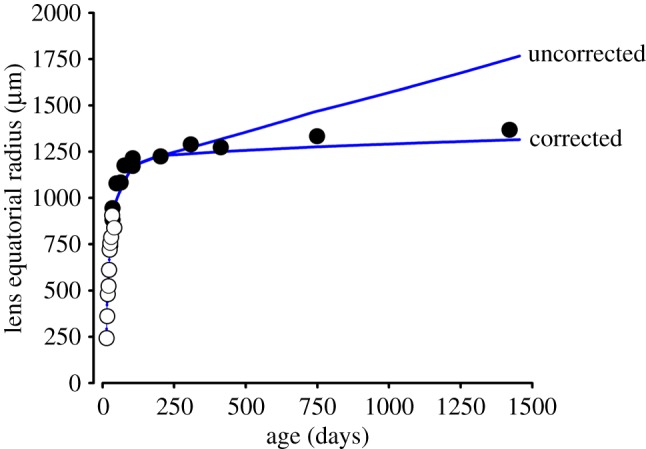
Growth in lens radius modelled across the lifespan. Empirical measurements were collected as described (open circles), or taken from the literature (closed circles) (7, 8). Growth simulations (blue lines) are shown with or without correction for fibre compaction (see the text for details).

The growth model consistently overestimated the lens radius at later ages (figure 6). For example, by the end of the lifespan, empirical measurements suggest that the lens radius is ≈1300 µm, whereas, a value of ≈1750 µm was predicted by the model. The size of newly formed fibres is known (figure 3) but there is little information on the fate of pre-existing cells located far below the lens surface. In the original model formulation [5], we assumed that mature fibre cells have a stable morphology. However, multiple lines of evidence (reviewed in [16]) indicate that over time cells in the lens core become compacted. If true, this provides a plausible explanation for the increasing discrepancy between model predictions and empirical measurements at later stages. Here, we used linear compaction factors of 0.030% and 0.025% to correct for loss of fibre volume in the periods six months to 2 years, and 2 to 4 years, respectively (figure 6 and appendix A). The rate of compaction is slow, but integrated over the lifespan, the effect is sizable. Expressed volumetrically, the uncompacted tissue would be twofold to threefold larger than actually observed. Thus, the lens would be expected to physically overgrow the eye in the absence of fibre cell compaction.

### 3.5. Cellular flow and flow reversal

The lens growth process is essentially one of cellular displacement. Cells introduced into the epithelium through mitosis in the proliferative zones force the egress of cells at the margin [4]. As a result, cells generally flow from higher to lower latitudes. Localized flow reversals can occur, however, in response to changes in zonal proliferation rate or *a*. Flow reversals can have the effect of isolating populations of epithelial cells and preventing their progeny from entering the fibre cell compartment.

To see how this operates, consider the relationships between CZ, PGZ and GZ. The proliferative zones, PGZ and GZ, produce a surfeit of cells, sufficient to fuel their own expansion (which occurs passively, in response to lens volume increase) while providing enough cells for export. By contrast, the CZ is mitotically inactive. Thus, expansion of the CZ in response to lens volume increase requires either spreading of constituent CZ cells or immigration of cells from the neighbouring mitotically active PGZ.

**Figure 7.**
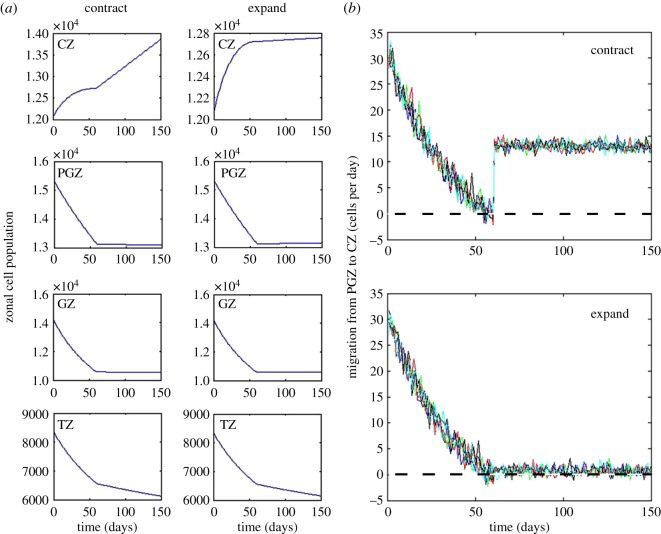
The projected effect of contraction or expansion in the footprint area of CZ cells (*a*_CZ_) on population dynamics in the period 60–150 days. Contraction of *a*_CZ_ results in a modest increase in the CZ cell population but has little effect on the populations of other zones (*a*). Monitoring the stochastic flow of cells from the PGZ into the CZ shows that by 60 days the net flow has fallen to zero (*b*). Results of five independent model runs are shown. Contraction of *a*_CZ_ from day 60 ensures a net positive flow of ≈15 cells day into the CZ. If *a*_CZ_ is allowed to expand in parallel to *a*_PGZ_, *a*_GZ_ and *a*_TZ_ (see appendix D), the net flow would remain close to zero with stochastic fluctuations ensuring that a small amount of reverse flow (CZ to PGZ) occurs.

We modelled the stochastic flow of cells across the CZ/PGZ border (figure 7). The flow was positive initially (i.e. cells tended to move from PGZ -> CZ) but, by three months, the net flow was zero, indicating that the rate of CZ cell spreading matched the rate of zonal expansion. Published data (appendix D) show that the footprint areas (*a*) of cells in the PGZ, GZ and TZ increase monotonically over the lifespan. By contrast, from 3 to 12 months, *a*_CZ_ decreases. To examine the consequences of such a decrease, lens growth was modelled under conditions where *a*_CZ_ was reduced in the period 3–12 months (CZ cell contraction case) or allowed to increase in parallel with the other zones (CZ cell expansion case, appendix D). The CZ cell population was modestly decreased in the expansion case because fewer cells could be accommodated within the zone but other population and growth parameters were largely unaffected (figure 7*a*). Although the zonal populations were not strongly influenced by changes in *a*_CZ_, there was a striking effect on the direction of flow at the CZ/PGZ border. If *a*_CZ_ was allowed to increase in parallel with the other zones, then the net daily flow of cells across the border was zero (figure 7*b*, lower panel). However, driven by stochastic fluctuations, each day a small number (less than 5) of CZ cells are expected to cross the border into the PGZ (and vice versa). Under conditions where *a*_CZ_ did not increase (i.e. the normal condition), the flow of cells was always strongly positive (figure 7b, upper panel). Thus, a consequence of the reduction in *a*_CZ_ during the period from 3 to 12 months was to temporarily corral cells in the central epithelium.

To examine the necessary conditions for cellular isolation, suppose that cells to be corralled are gathered in a zone which covers an area (or a volume) *A*(*t*) at time *t*, and contains *X*(*t*) cells of individual size *a*(*t*) with offspring branching mechanism *Z*. At time *t*, *X*(*t*) cells produce a total of *O*(*t*) offspring cells. It follows that at time *t* + 1 there will be no cellular emigration if and only if

$$\begin{array}{rl} O(t)\cdot a(t+1) & \leq A(t+1),\text{ which in turn is equivalent to}\\ & \;O(t)\cdot a(t+1)-X(t)\cdot a(t)\leq A(t+1)-A(t). \end{array}$$

Considering this condition ‘in the mean’ we obtain

(E(Z)a(t+1)−a(t))E(X(t))≤E(A(t+1))−E(A(t)).

Assuming that the system also grows ‘in the mean’, i.e. that the right-hand side is positive, one sufficient condition to ensure ‘isolation’ is to require

E(Z)a(t+1)−a(t)=0.

This condition depends solely on cellular proliferation (distribution of *Z*) and individual cell sizes (*a*(*t*)). If *a*(*t* + 1) > *a*(*t*), we need to lower proliferation rates sufficiently to satisfy the equation above. If, as in our case, there is no proliferation (i.e. *E*(*Z*) = 1), then cells can be held in the quarantine zone by dropping the growth rate of *a*(*t*) to zero (i.e. to have *a*(*t* + 1) = *a*(*t*)), which is what happens in the CZ in the period 3–12 months.

### 3.6. Achieving precision with a stochastic growth engine

Simulations suggest that the ocular lens, a precisely defined biological structure, can be generated using a stochastic engine (figures 4 and 6). Even after more than 1000 iterations, each involving tens of thousands cells acting randomly and independently, the precision of the lens growth process is striking. How might this arise? There appear to be two key elements. First, before E14, the system grows deterministically (but simply, because cells appear to multiply as fast as they can). After E14, we have a fully stochastic mechanism, but by that phase the lens epithelium contains more than 10 000 cells. That number is already sufficiently large for the law of large numbers to operate. This alone would confer a relatively high degree of precision but the effect works in tandem with a second phenomenon. Azevedo & Leroi [6] have shown that the *coefficient of variation* (CV), where

$$\text{CV}=\frac{\text{standard deviation}}{\text{mean}},$$

is a feature of organ growth [6]. Suppose that an organ is built using a standard branching process (*X*(*t*)), where

$$X(t+1)=\overset{X(t)}{\underset{i=1}{\sum }}Z_{i},$$

and (*Z_i_*) is a family of iid integer-valued random variables whose distributions are given by the branching mechanism

$$Z∼\left(\begin{array}{ccccc} 0 & 1 & 2 & 3 & \cdots \\ p_{0} & p_{1} & p_{2} & p_{3} & \cdots \end{array}\right).$$

If we start with *N* cells, it is not difficult to calculate CV at time *t*;

$$\text{C}{\text{V}}_{t}=\sqrt{\frac{\text{Var(}Z\text{) }}{E(Z)[E(Z)-1]}\cdot }\frac{1}{\sqrt{N}}\sqrt{1-\frac{1}{{\left[E(Z)\right]}^{t}}}.$$

In our model, the proliferative zones (GZ and PGZ) are flanked by mitotically quiescent regions (CZ and the fibre cell compartment) offering a pathway by which supernumerary cells can escape the growth process. This arrangement improves the precision of the process further, by introducing a correction term to the *Azevedo-Leroi* CV (for calculations and mathematical details see appendix E).

The effect of non-mitotic zones is that there exists a factor, say *λ*, such that emigration can be represented as (1 − *λ*)(*O*(*t*) − *X*(*t*)). We consider a constant H:=λE(Z)+(1−λ); observe 1 < *H *< *E*(*Z*) in our situation. The CV for such a process, say ${\text{CV}}_{t}^{\lambda }$ is given by

$${\text{CV}}_{t}^{\lambda }=\lambda \sqrt{\frac{\text{Var(}Z\text{) }}{H(H-1)}}\cdot \frac{1}{\sqrt{N}}\cdot \sqrt{1-\frac{1}{H^{t}}}.$$

It follows that

$$0<\frac{{\text{CV}}_{t}^{\lambda }}{\text{C}{\text{V}}_{t}}=\sqrt{\frac{E(Z)}{E(Z)+1/\lambda -1}}\cdot \frac{\sqrt{1-1/H^{t}}}{\sqrt{1-1/{\left[E(Z)\right]}^{t}}}<\sqrt{\frac{E(Z)}{E(Z)+1/\lambda -1}}<1.$$

In our case, we typically have *E*(*Z*) close to 1, implying that the factor $\sqrt{E(Z)/E(Z)+(1/\lambda )-1}$ is close to $\sqrt{\lambda }$, i.e.

$${\text{CV}}_{t}^{\lambda }\leq \text{const}\cdot \sqrt{\lambda }\cdot \text{C}{\text{V}}_{t}.$$

Hence, a zonal structure improves precision with factor $\sqrt{\lambda }$. For the lens process, *λ* changes with time and is mostly of the order 1/10. It follows that if a non-zonal arrangement were to achieve (via the law of large numbers, for example) a relative precision of ±5%, then the introduction of a zonal structure would further improve the precision to around ±1%. This calculation applies to any organ-building stochastic system where an active growth region is flanked by non-mitotic zones into which excess cells may escape.

### 3.7. Cluster size and transit time

Our zonal model postulates that cells are displaced from the epithelium through the mitotic activity of cells located at higher latitudes. In the young lens, where all cells are dividing, it is possible that given sufficient time, even cells located near the apical pole could be displaced into the fibre compartment. Later, as the zonal structure is established, only the PGZ and GZ contain mitotic cells. The progeny of cells marooned in the CZ are unlikely to differentiate into fibre cells (figure 7). PGZ cells, however, will be gradually displaced towards the lens equator, slowly traversing the PGZ and then the GZ. Cells probably accelerate as they draw nearer the equator, owing to the increasing cumulative ‘push’ imparted by mitosis occurring at higher latitudes [4]. Cells are likely to undergo multiple cell divisions as they cross the PGZ and GZ. Indeed, lineage tracing experiments have identified clusters of clonally related cells near the epithelial margin [17]. But what is the size distribution of emergent clusters, how will this depend on age, and how long does it take for clusters to complete their migration and enter the body of the lens?

In appendix F, we develop a model that traces the path of an epithelial cell as it travels from higher to lower latitudes. We obtain the following basic formula (formula (F 9) from appendix F; where *L*_0_ is the starting layer of the travelling cell, *L* is the final layer, *N*_∞_ is the travelling time and *p_k_* is the proliferation rate during the *k*th day):

$$\overset{N_{\infty }}{\underset{k=1}{\prod }}(1+p_{k})=\frac{L}{L_{0}}.$$

After *N* cycles the original cell, which started on layer level *L*, would on average reach level *L_N_*. Using the Markov property, we obtain

$$L_{N}=L\overset{N}{\underset{k=1}{\prod }}(1+p_{k}).$$

Because we know the number of levels, say *T*, then by solving the equation *L_N_* = *T*, we obtain an estimate for the average number of cycles, *N*, required to reach the equator. Interestingly, the average cluster size does not change if we change *p_k_* values (see formula (F 8) in appendix F); for the mouse lens, we predict clusters consisting of around 50 cells on average. The intuition is that although a higher proliferation rate produces more new cells sooner, it also speeds the cluster in the direction of the equator. Although clone size is not sensitive to proliferation rate, transit time is (see formula (F 10) in appendix F).

## 4. Conclusion

Previously, we developed a stochastic branching process-based model of lens growth [5]. The model was formulated from a simple set of axioms and we used it to explore how the unique geometry of the lens influences its growth. We restricted the model to a narrow time frame, so that we could make the simplifying assumption that growth parameters remained constant. In the present study, we modelled growth over the entire lifespan. This necessitated the incorporation of new empirical data on parameters such as age-dependent changes in cellular proliferation rate, fibre cell dimensions and cell-cycle duration. Output of the model was consistent with experimental measurements of cell population dynamics and radial growth [4] although, at later time points, a radial correction factor had to be introduced to compensate for the presumed (but unproven) effects of fibre cell compaction.

### 4.1. Three distinct phases of lens growth

Based on our modelling efforts, we can now distinguish three growth phases: early (from lens formation on E10.5 to eyes opening at P14), middle (from two weeks to six months of age) and late (from six months to the end of life).

Early growth is explosive. In mice, eyes open at two weeks, so the lens must be built quickly. This requires the production of a large number of cells in a short period. Consequently, all cells are cycling and the cycle is short. A temporary capillary network, the *tunica vasculosa lentis*, envelops the embryonic lens and this may be necessary to support rapid growth. The footprints of individual epithelial cells are small (perhaps because there is insufficient time between cycles for robust cellular growth). As a result, proliferation zones are densely populated, maximizing cell production. Any shortfall in cell production at this stage has a permanent detrimental effect on lens size.

In the middle period, the zonal structure is well developed but there is a risk of overproduction of cells because the growth in epithelial surface area (with a constant *η*) means that more and more cells are located within the proliferative zones. Exponential overgrowth is avoided by a generalized cell spreading phenomenon, which restricts population increase in the GZ and PGZ and consequently serves as a powerful governor on lens growth. The movement of cells from the CZ into the fibre cell compartment is blocked temporarily by a decrease in *a*_CZ_, which reverses the stochastic flow at the CZ/PGZ border, leaving CZ cells marooned in the pupil space. Migrating cells undergo multiple divisions as they traverse the proliferation zones, leading to the formation of clonal clusters. Migration times vary from 50 days at the beginning of this period to more than 400 days by the end.

Late lens growth is characterized by the slow but steady deposition of fibre cells at the lens surface. Cell spreading is completed, but declining proliferation rates in the GZ and PGZ limit the possibility of exponential growth. Late phase growth lasts for several years (at least in laboratory settings) and although the addition of cells is slow, significant radial growth over this extended period is expected. Here, the phenomenon of fibre compaction is important, contributing to the internal refractive properties of the lens [16] and preventing the lens from physically overgrowing the eyeball.

### 4.2. Proliferation rate and Δ*a* are key determinants of lens growth

An important finding from this work is that lens growth dynamics can be modelled adequately by merely adjusting proliferation rates and footprint areas of individual epithelial cells. Although this effect is not readily apparent in equations wherein all four zones are combined, it can be appreciated in the simplified case, where only one zone exists (this corresponds, for example, to the situation at E14.5, when S-phase cells are distributed uniformly throughout the epithelium). The formulae are provided in appendix C (see equation (C 2)), where we show that the average number of cells in the epithelium will increase, remain the same, or decrease, based solely on whether

r⋅2ρw>Δa,orr⋅2ρw=Δa,orr⋅2ρw<Δa,

where r:=r(t) is the proliferation rate and Δa:=a(t+1)−a(t) is the footprint area increment. Because the cross-sectional size of the fibre cells (*ρw*) is constant from six months onward (figure 3), it is noteworthy that such a ‘macroscopic effect’ (i.e. total number of cells in the epithelium) is determined by the ‘microscopic features’ *r* and Δ*a*. Conceptually, we envisage the contributions of *r* and Δ*a* as two pedals in an automobile, one serving as the accelerator, the other as the brake. Thus, the entire growth process is achieved by gently applying pressure on one or other pedal. It is not yet clear whether the ‘pedals’ have biological identities, but they could be analogous to the Hippo and TOR pathways which, in other organ systems, regulate organ growth by controlling cell number and cell size, respectively [18]. In support of this notion, it was recently shown that inactivation of PI3 K, an upstream regulator of mTOR, results in a lens that is too small but otherwise histologically normal [19].

In our model, cell density and local cell proliferation rates (key determinants of lens growth) are treated as independent variables. In the absence of evidence to the contrary, this seems the most parsimonious approach. The model also assumes that cells flow towards the edge of the epithelium in response to mitotic pressure generated through cell division at higher latitudes. There is some experimental support for this view. For example, in hypophysectomized frogs, lens epithelial cell division is completely blocked. This results in the immediate cessation of fibre cell formation, as expected if cell division were the primary driver of cell migration [9]. We note, however, that other authors [20] have measured cell density distributions across the lens epithelium and related the shape of the density curves to the production and egress of cells (the ‘ordered pull-through model’ (OPT)). In the OPT model, density and proliferation are not treated as independent parameters. Rather, cell density is proposed to be responsive to both a ‘push’, provided by epithelial cell production, and a ‘pull’, resulting from the flow of cells into the fibre compartment. The two lens growth models are not mutually exclusive and, in either case, further experimental data, perhaps on the nature of forces acting on the migrating cells, will be required to evaluate them properly.

### 4.3. Absence of ‘catch-up’ growth

**Figure 8.**
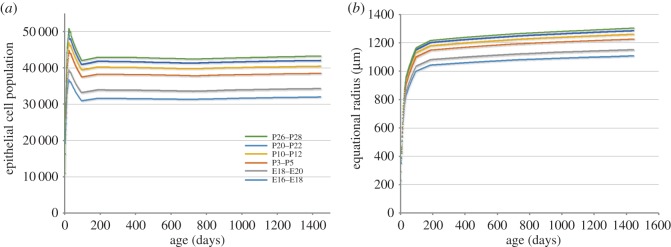
Effect of a 2-day hiatus in cell proliferation on the epithelial cell population (*a*) and radial growth (*b*) of the lens. Note the significant reduction in epithelial population and lens radius when growth is interrupted early (E16–E18) in development. A 2-day pause later in development (P26–P28) has little effect on lens growth (compare with figures 4 and 6).

Typically, the growth of organisms and organs ceases at maturity. Once adulthood is reached, cell proliferation rates are reduced to balance cell death rates. In that sense, lens growth is unusual because despite the absence of cell death, cells continue to be produced until the very end of life [21]. The eyeball itself stops growing relatively early in postnatal development [22]. Thus, a structure with indeterminate growth (the lens) is housed within a receptacle (the eye ball) with a determinate growth mode. Perhaps because growth is indeterminate, the lens shows no sign of ‘catch up growth’. If the growth zones of the lens are not adequately populated early in development, the resulting growth deficit appears to be permanent. For example, inactivation of the gap junction gene *Gja8* causes a temporary reduction in epithelial cell proliferation in P2 mice [23]. Although brief, the interruption is sufficient to cause a lifelong growth deficit. A similar effect was noted following conditional deletion of the p110α subunit of phosphoinositide 3-kinase [19]. We replicated this effect with our model (figure 8) and found that a two-day hiatus in proliferation is particularly consequential if it occurs in the young lens.

Cases of microphakia are not uncommon in ophthalmology but there are no reported cases of macrophakia (i.e. an overly large lens), implying that either growth rates are already maximal or that the lens actively compensates for cellular overproduction (for example, by adjusting the fibre cell compaction factor). In mice, lens growth defects often lead to microphthalmia [19,23] suggesting that at least with regard to growth, the eye takes its cue from the lens.

### 4.4. Clones, transit times and cataract

Our calculations indicate that cells are likely to undergo multiple cell divisions as they traverse the proliferation zones. The calculations suppose that the movement of cells is vectorial, and that there are no cellular eddies that might cause a cell to dwell at a particular latitude (details are given in appendix F). The expected maximal clone size of less than 100 cells is consistent with the results of lineage tracing experiments [17]. A counterintuitive finding was that cluster size was not influenced strongly by proliferation rates and that, as a result, clusters of similar size are expected to form in young lenses (where proliferation rates are high) and old lenses (where rates are low). By contrast, proliferation rate had a powerful effect on transit time. For example, progeny of a PGZ cell located close to the CZ/PGZ border at P14 would take 7 weeks to differentiate into fibre cells. For cells at the equivalent location in a six-month-old lens, the expected transit time is more than 13 months.

Deep sequencing studies have recently identified somatic sequence variants in DNA from human lens epithelial cells. The measured allele frequencies imply the presence of millimetre-sized clones [24]. Sunlight-exposed cells are prone to somatic mutations and UV-B exposure is a risk factor for cataract [25]. Cellular flow from the epithelium into the body of the lens provides a route by which mutations generated in central, sun-exposed regions might be amplified and conveyed to the fibre cell mass. If mutations were to occur in genes necessary for fibre cell transparency, they might manifest as spoke-like opacities in the body of the lens (each spoke corresponding to a cluster of mutant fibres derived from a single, sun-exposed epithelial antecedent). In the mouse, cells appear to be temporarily quarantined in the central epithelium (figure 6) and only released at stage when the projected transit time exceeds the remaining lifespan. It will be interesting to determine if similar mechanisms operate in humans and whether they serve to suppress cataract formation until late in the lifespan.

## Supplementary Material

MATLAB code for running the full lifespan lens growth model

## Figures and Tables

**Figure 9.**
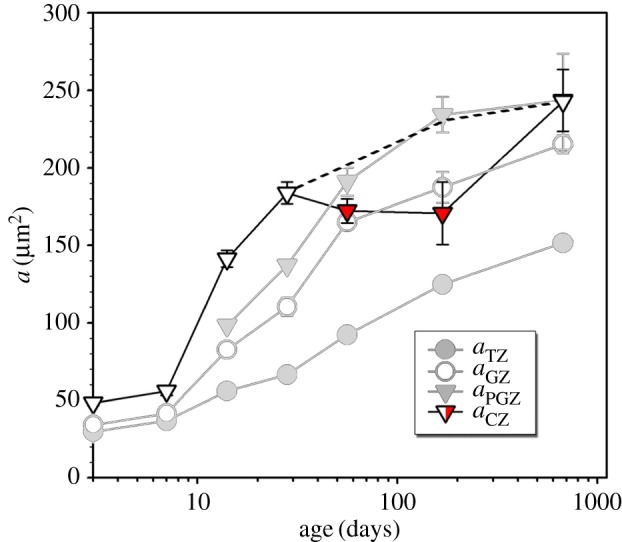
Expansion in cellular footprint area (*a*) of epithelial cells in the four zones (CZ, PGZ, GZ and TZ) of the lens epithelium during postnatal development (data adapted from [4]). Footprint areas increase monotonically in PGZ, GZ, and TZ. However, CZ footprint area decreases slightly (red) in the period 2 months (60 days) to 6 months (180 days), before resuming its expansion at later time points. The dashed line indicates growth behavior if the expansion of the CZ was adjusted to match that of the other zones (see figure 7*b* of the current manuscript).

## Data accessibility

The Matlab code required to run the growth model has been uploaded as part of the electronic supplementary material.

## Appendix A. Model description

The axioms underlying the model have been described [5]. In this paper, we allow for cellular dynamics to change with time and also introduce compaction factors which act on the lens late in the lifespan. Consequently, the full lifespan model is more complex, requiring us to adjust the corresponding formulae accordingly.

Consider some time *t* in the life of a mouse. The lens epithelium has a surface area of *A*(*t*) µm^2^ and (in principle) is divided into four zones, where index 1 corresponds to CZ, index 2 to PGZ, index 3 to GZ, index 4 to TZ. The surface areas of each of the zones are presented via formulae *A_i_*(*t*) = *η_i_*(*t*)*A*(*t*), *i *= 1, 2, 3, 4. Observe that we allow for some ‘*i*’ to have $\eta_{i}(t)=0(\Leftrightarrow A_{i}(t)=0)$ ; meaning simply that up to time *t*, the *i*th zone has not yet developed. The number of cells in zone ‘*i*’ is *X_i_*(*t*), and the surface area covered by a single cell is *a_i_*(*t*) Hence, *A_i_*(*t*) = *X_i_*(*t*)*a_i_*(*t*). Let us describe what happens between *t* and *t* + 1.

The nature of the TZ is such that cells within TZ do not divide. In principle, we allow for all other cells to divide, remain the same or die in the other three zones, i.e. we consider three probability distributions of the form

$$Z_{i}(t)∼\left(\begin{array}{ccc} 0 & 1 & 2\\ p_{0}^{i}(t) & p_{1}^{i}(t) & p_{2}^{i}(t) \end{array}\right),$$

and then develop the model based on the specifics of actual data on *Z_i_*(*t*). In the case of the mouse lens we have, $p_{1}^{1}(t)\equiv 1,p_{0}^{2}(t)\equiv 0\equiv p_{0}^{3}(t)$ (see also [4] and [5]). Hence, the information about *Z_i_*(*t*) is in essence $q(t):=p_{2}^{2}(t)$ and $r(t):=p_{2}^{3}(t)$ (observe that $p_{1}^{2}(t)=1-q(t)$ and $p_{1}^{3}(t)=1-r(t)$). Assuming that $\left(Z_{2,n}(t):n\in N\right)$ are iid copies of *Z*_2_(*t*) and $\left(Z_{3,n}(t):n\in N\right)$ are iid copies of *Z*_3_(*t*), we obtain the total number of offspring cells in the PGZ and GZ between *t* and *t* + 1:

$$\begin{array}{rl} O_{2}(t) & :=\overset{X_{2}(t)}{\underset{n=1}{\sum }}Z_{2,n}\\ O_{3}(t) & :=\overset{X_{3}(t)}{\underset{n=1}{\sum }}Z_{3,n}. \end{array}$$

The interior of the lens consists of elongated fibre cells with hexagonal equatorial plane intersections given by the length *ρ*(*t*) and width *w*(*t*) of hexagons [5]. In the full lifespan model, we also allow for a compaction effect. Because the compaction phenomenon is not yet well understood, we assume only that it has some global effect on the radius of the lens *R*(*t*). The measure of that effect is given by the compaction factor Δ = the percentage of radius that has contracted (0 ≤ Δ < 1, but in general Δ is either 0 or very small ≈10^−4^). The consequence is that the offspring cells should not only cover the increase in growth but also the effect of the compaction process. Let us denote by *v* the number of cells required to offset the effect of the compaction. Based on the geometry of the lens we obtain that

$$2\rho (t)w(t)\cdot v=A(t)-2\pi {\left[R(t)(1-\Delta )\right]}^{2}=A(t)[1-{\left(1-\Delta \right)}^{2}]=A(t)\Delta (2-\Delta ).$$

Hence, we obtain that

A 1 $$v=\frac{A(t)}{2\rho (t)w(t)}\Delta (2-\Delta ).$$

We denote by *x* = the number of cells that move from PGZ to CZ, *y* = the number of cells that move from PGZ to GZ, *z* = the number of cells that move from GZ to TZ, *u* = the number of cells that move from TZ to the fibre compartment and are needed to maintain the increase in growth. Observe that the total number of cells that move from the epithelium to the fibre compartment is *u* + *v*. The basic immigration–emigration process now follows the one described previously [5]:

A 2 $$\begin{array}{rl} X_{1}(t+1) & =X_{1}(t)+x,\\ X_{2}(t+1) & =O_{2}(t)-x-y,\\ X_{3}(t+1) & =O_{3}(t)+y-z\\ \text{and}\;X_{4}(t+1) & =X_{4}(t)+z-(u+v). \end{array}\}$$

As *v* is given in (A 1), we again have four equations with four unknowns, but the dynamics are now different, because we allow for changes in time and have the additional factor Δ. Nevertheless, it is not difficult to solve the equations, and we obtain the following solutions. Observe first that, for every *i* = 1, 2, 3, 4, we have

$$\begin{array}{rl} \frac{X_{i}(t+1)a_{i}(t+1)}{\eta_{i}(t+1)} & =A(t+1)=2\rho (t+1)w(t+1)u+A(t)\\ & =2\rho (t+1)w(t+1)u+\frac{X_{i}(t)a_{i}(t)}{\eta_{i}(t)}. \end{array}$$

It follows that, for every *i* = 1, 2, 3, 4,

$$X_{i}(t)-X_{i}(t+1)=-\frac{2\rho (t+1)w(t+1)}{a_{i}(t+1)}u+X_{i}(t)\left[1-\frac{\eta_{i}(t+1)}{\eta_{i}(t)}\cdot \frac{a_{i}(t)}{a_{i}(t+1)}\right].$$

Using (A 1), we now obtain the complete solution of (A 2):

A 3 $$\begin{array}{rl} v & =\frac{A(t)}{2\rho (t)w(t)}\Delta (2-\Delta ),\\ u & =\frac{1}{1+\sum_{i=1}^{4}E_{i}(t)}\left[\overset{4}{\underset{i=1}{\sum }}F_{i}(t)X_{i}(t)+\overset{3}{\underset{i=2}{\sum }}(O_{i}(t)-X_{i}(t))-v\right],\\ x & =E_{1}(t)u-F_{1}(t)X_{1}(t),\\ y & =(1+E_{3}(t)+E_{4}(t))u-F_{3}(t)X_{3}(t)-F_{4}(t)X_{4}(t)-(O_{3}(t)-X_{3}(t))+v,\\ \text{and}\;z & =(1+E_{4}(t))u-F_{4}(t)X_{4}(t)+v, \end{array}\}$$

where *E_i_*(*t*) and *F_i_*(*t*) are deterministic and given by

$$\begin{array}{rl} E_{i}(t) & =\frac{2\rho (t+1)w(t+1)}{a_{i}(t+1)}\\ \text{and}\;F_{i}(t) & =1-\frac{\eta_{i}(t+1)}{\eta_{i}(t)}\cdot \frac{a_{i}(t)}{a_{i}(t+1)}. \end{array}\}$$

Observe that for $\eta_{i}(t+1)=\eta_{i}(t)$ and $a_{i}(t+1)=a_{i}(t)$ we get *F_i_*(*t*) = 0. For $\Delta =0,\eta_{i}(t+1)=\eta_{i}(t)$ and ρ(t+1)=ρ(t),w(t+1)=w(t), solutions (A 3) correspond to the solution given in [5]. In particular, *E_i_*(*t*) and *F_i_*(*t*) (respectively), correspond to *C_i_*(*t*) and *D_i_*(*t*). Observe also that, because 0 ≤ Δ < 1, we have *v* = 0 if and only if Δ = 0.

## Appendix B. Cell-cycle estimates

In adult lenses, cell-cycle time is approximately 24 h, with a 12 h S-phase [13]. However, a simple assessment of the increase in cell number during embryonic lens development indicates that the lens cell-cycle duration must be shorter at earlier stages. For example, at E10.5, the lens is a hollow vesicle consisting of some 1200 cells. Of these, only half (those covering the anterior lens surface) are mitotically active after E12.5. By E14.5, we estimate that the epithelium alone contains 11 000 cells (and the interior almost twice that many). Clearly, even if all the original 600 anterior cells were actively cycling throughout the intervening period, it would be impossible to generate the necessary number of cells in the four 24 h cycles between E10.5 and E14.5.

To obtain estimates of cell-cycle lengths in the embryonic lens, we present a simple model relating the observed proliferation rates of cells to their cell cycle. Consider a set of cells within an organism, observed at a particular stage in the life of the organism and at a particular position within the organism. First, we postulate that, at this stage and at this position, the set consists of cells, which are ‘cell-cycle homogeneous’, in the following sense.

*Postulate 1.* All cells within the observed set have a cell cycle of the same length. Measured in hours, the length of the cell cycle is *C*.
*Postulate 2.* All cells within the observed set have the same ‘probability of proliferation within *C* hours’; denoted by 0 < *p* < 1. More precisely, if a cell is selected randomly from the set, then the probability that the cell will start dividing within the next *C* hours is *p*.
*Postulate 3.* There exists a fixed number 0 < *α* < 1, such that the S-phase for each cell within the observed set lasts *αC* hours.

Second, we assume that we can perform an ‘S-phase labelling’ experiment on such a ‘cell cycle homogeneous’ set. Thus, for *t* hours, we expose the set of cells to a label that will be taken up by any cell, which is in the S-phase during any part of the *t*-hour period. We also assume that our experiment can be performed on a sample of ‘cell cycle homogeneous’ sets (with the same *C*, *p*, *α* for all elements of the sample).

Third, our experimental procedure should satisfy the following assumptions:

(a1) Relative frequencies of the number of labelled cells converge to a fixed number 0 < *r* < 1.
(a2) *αC* + *t* < *C*.
(a3) The starting times of the S-phases are distributed uniformly within a time interval of C hours.

*Remark.* (a1) is a standard statistical assumption on the stability of relative frequencies; without it most statistical inference studies would lack meaning. (a2) ensures that the *t*-interval is short enough that our analysis remains ‘within a single cycle’. (a3) is a standard initial assumption if we have no additional information about cycle properties.

Using our postulates and assumptions, we conclude that *r* is with respect to *αC* + *t* as *p* is with respect to *C*, i.e. we obtain our *basic formula*

B 1 $$\frac{r}{\alpha C+t}=\frac{p}{C}.$$

Observe that (B 1) and (a2) imply

B 2 $$\begin{array}{rl} & \alpha p<r<p, \end{array}$$

B 3 $$\begin{array}{rl} & r\to \alpha p\Rightarrow t\to 0\;\mathrm{or}\;M\to \infty \end{array}$$

B 4 $$\begin{array}{rl} \text{and}\; & r\to p\Rightarrow \alpha C+t\to C. \end{array}$$

Simply stated, (B 3) and (B 4) show that although it is mathematically possible to consider *r* to be close either to *αp* or to *p*, it is not realistic to have such relationships in real-life situations.

Observe that there are five variables in (B 1), so having some knowledge about any of them increases the usefulness of formula (B 1). Typically, we expect to know *t*, because we control the experiment. In our experiments, we always have *t* = 1 h. In many cases, the literature on cell-cycle properties provides evidence strong enough to estimate α. In our studies, we have assumed that from E14 on in the life of mouse *α* = 1/2 (we think that our evidence shows that between E10 and E14 this assumption does not hold, but we intend to devote a separate article to the details of this very early stage of the lens development).

Under assumptions *t* = 1 and α = 1/2, formula (A 1) leads to the following:

B 5 $$p=\frac{r}{1/2+1/C}$$

and

B 6 $$C=\frac{1}{r/p-1/2}.$$

Observe that the estimate for quantity *r* is given via direct measurements of the results of our experiment.

Let us take the example of the E14–E16 time period to illustrate how to estimate *C* in actual situations. Consider a realistic estimate of $6^{h}\leq C\leq 24^{h}$ (for smaller or larger *C*, the results are even further from actual measurements). Our measurements give us *r* ≈ 0.48 at E14.5. Using (B 5), we obtain that *p* (now as a function of *C*) gives

$$\begin{array}{rl} C & =6^{h}\Rightarrow p\approx 0.75\\ C & =24^{h}\Rightarrow p<1 \end{array}$$

and we shall go with the ‘worst case scenario’ and take *p* = 0.75 for *C* = 6*^h^* and *p* = 1 for *C* = 24*^h^*. Observe that E14–E16 consists of 48*^h^*, so with *C* = 6 we follow eight cycles, while with *C* = 24 we have two cycles. Furthermore, according to our measurements the radius of the spherical lens is

B 7 $$\begin{array}{rl} R & =223\;\text{μ}m\;\text{at}\;E14.5\\ \text{and}\;R & =350\;\text{μ}m\;\text{at}\;E16.5. \end{array}\}$$

We employ our model as developed [5]. Our data show that at this stage the entire epithelium acts as a single GZ with a proliferation rate *p*, that a single cell covers on average 30 µm^2^, and that equatorial hexagonal intersections have an area of 7 µm^2^. Let us provide a brief sketch of average changes according to our model. Consider a single cycle and denote by *X*_0_ the initial number of epithelial cells and by *X*_1_ the number of epithelial cells at the end of the cycle (we use expected values, i.e. ‘averages’ for this calculation). Following our model, we have that Δ(epithelial area)=2Δ(equatorial area). Hence, if we denote by *X*_∞_ the number of cells that leave the epithelium we obtain

$$[(1+p)X_{0}-X_{\infty }-X_{0}]\cdot 30=2\cdot 7\cdot X_{\infty }$$

which leads to

$$X_{1}=\left(1+\frac{7}{22}p\right)X_{0}.$$

This implies that in a single cycle the radius of the lens will on average change with the factor

B 8 $$\sqrt{1+\frac{7}{22}p}.$$

For *p* = 0.75, this gives the factor 1.1129 and after eight cycles we obtain the radius estimate at E16 to be around 525 µm (instead of measured value 350 µm). For *p* = 1, the factor (B 8) is 1.1481 and after two cycles we obtain the E16 radius of 294 µm (instead of measured value 350 µm).

Hence, we end up with *C* ≈ 12*^h^* (i.e. four cycles within E14–E16 time period). We interpolate linearly for the values of *r* in between (we have detailed measurements for *r* at E14.5 and E16.5) and use (B 5) to obtain *p*.

## Appendix C. The relationship between *r* (proliferation rate) and Δ*a* (change in the area of an individual cell footprint) determines whether the epithelial population will increase or decrease.

During embryonic development, the lens epithelium acts as a single GZ. During this phase, it is easier to appreciate the relationships between key parameters that regulate the growth (on average) of the number of epithelial cells. The same effect occurs within the four zone structure, but there the formulae are more complicated. We consider a single cycle time period from *t* to *t* + 1 and introduce the following notation:

$$\begin{array}{rl} m(t) & :=E(X(t))=E(X_{3}(t)),\\ \rho & :=\rho (t+1)=\rho (t),\\ w & :=w(t+1)=w(t),\\ a & :=a(t),\\ a+\Delta a & :=a(t+1),\\ r & :=r(t)\\ \text{and}\;u & :=(X_{E\infty }). \end{array}$$

As Δ(surface area)=2Δ(equatorial area), we obtain

2ρwu=m(t+1)(a+Δa)−m(t)a.

Furthermore,

m(t+1)=(1+r)m(t)−u.

This enables us to calculate *u* in terms of *m*(*t*), i.e.

2ρwu=[(1+r)m(t)−u](a+Δa)−m(t)a=rm(t)(a+Δa)+m(t)Δa−u(a+Δa),

which gives

$$u=m(t)\cdot \frac{r(a+\Delta a)+\Delta a}{2\rho w+(a+\Delta a)}.$$

Therefore, we can explicitly express *m*(*t* + 1) in terms of *m*(*t*):

C 1 $$m(t+1)=m(t)\left[(1+r)-\frac{r(a+\Delta a)+\Delta a}{2\rho w+(a+\Delta a)}\right]=m(t)\cdot \frac{(2\rho w+a)+2\rho wr}{(2\rho w+a)+\Delta a}.$$

Hence,

C 2 $$\begin{array}{rl} & m(t+1)>m(t)\Leftrightarrow r\cdot 2\rho w>\Delta a\\ \text{and}\; & m(t+1)=m(t)\Leftrightarrow r\cdot 2\rho w=\Delta a. \end{array}\}$$

Our data show that during most of the prenatal period $\rho w\approx 7\text{μ}{\text{m}}^{2}$, which implies that the ‘threshold point’ between *r* and Δ*a* is 14*r* = Δ*a*.

This shows how proliferation rate (denoted here by *r*) and change in the surface area of individual epithelial cells (Δ*a*), acting together may regulate the growth process. If 14*r* ≈ Δ*a*, then the growth process will maintain a stable number of cells in the epithelium. If 14*r* > Δ*a*, then the number of cells in the epithelium will increase. However, if we enlarge Δ*a* so that it is bigger than 14*r*, there will be a decrease in the number of epithelial cells.

As an illustration, observe the time period around the birth of the mouse. At E20 we have *r* = 0.23, i.e. 14*r* = 3.22. This means that we need to have Δ*a* < 3.22 µm^2^ if we want to keep the growth of *X*(*t*). Interestingly, it is after P1 that we observe a more significant change in Δ*a* (it starts to get bigger).

## Appendix E. Coefficient of variation

Azevedo & Leroi [6] introduced the CV as an important element in the study of organ growth. The CV, given by

$$\text{CV}=\frac{\text{standard deviation}}{\text{mean}},$$

provides us with the measure of the relative size of a dispersion. We show here how our model leads to the realization that the movement of some epithelial cells into the CZ and the fibre compartment reduces the CV and improves the precision of the organ growth process.

*Wald Formula*. Suppose that $\left\{X;Z_{i}:i\in N\right\}$ is a family of independent random variables with values in $N_{0}=N\cup^{\{}0\}$. Suppose also that the family {*Z_i_*} consists of identically distributed random variables with law

E 1 $$Z∼\left(\begin{array}{ccccc} 0 & 1 & 2 & 3 & \cdots \\ p_{0} & p_{1} & p_{2} & p_{3} & \cdots \end{array}\right).$$

Consider a random variable *O* (*O* stands for offspring) defined by

E 2 $$O:=\overset{X}{\underset{i=1}{\sum }}Z_{i}.$$

Using Wald's technique, i.e. splitting into partition sets (X=n),n∈N∪{0}, one obtains several moment identities

E 3 $$\begin{array}{rl} E(O) & =E(Z)E(X)\\ \text{and}\;E(X\cdot O) & =E(Z)E(X^{2}) \end{array}\}$$

and

E 4 $$\text{Var(}O\text{)}=\text{Var(}Z\text{)}E(X)+{\left[E(Z)\right]}^{2}\text{Var(}X\text{).}$$

Suppose now that we have two sums of type (E 2) and that ‘counting variables’ are heavily dependent. More precisely, consider

E 5 $$W∼\left(\begin{array}{ccccc} 0 & 1 & 2 & 3 & \cdots \\ q_{0} & q_{1} & q_{2} & q_{3} & \cdots \end{array}\right),$$

and a deterministic function $h:N_{0}\to N_{0}$, with *h*(0) = 0. Suppose that we have a family $\left\{X;Z_{i};W_{i}:i\in N\right\}$ of independent random variables such that, for every $i\in N,Z_{i}∼Z,W_{i}∼W$, and a random variable *Y* = *h*(*X*). Consider *O*, defined by (E 2), and $\tilde{O}$, defined by,

E 6 $$\tilde{O}:=\overset{Y}{\underset{i=1}{\sum }}W_{i}.$$

Using Wald's technique, it is easy to see that

E 7 $$E(O\cdot \tilde{O})=\overset{\infty }{\underset{n=1}{\sum }}P(X=n)E\left[\overset{n}{\underset{i=1}{\sum }}\overset{h(n)}{\underset{j=1}{\sum }}Z_{i}W_{j}\right]=E(Z)E(W)\overset{\infty }{\underset{n=1}{\sum }}P(X=n)n\cdot h(n)=E(Z)E(W)E(X\cdot h(X)).$$

*Single Zone Epithelium*. We treat this case separately for two reasons. First, this is the case which describes the prenatal mouse lens. Second, owing to the simplicity of the formulae involved, it is easier to appreciate the contribution in this case than in the general case.

Suppose that the epithelium consists of *X* cells with footprint area *a* and a proliferation law

$$Z∼\left(\begin{array}{ccc} 0 & 1 & 2\\ 0 & 1-p & p \end{array}\right);$$

i.e. *E*(*Z*) = 1 + *p *> 1. A single cell cycle is modelled via a family $\left\{X;Z_{i}:i\in N\right\}$ of independent random variables with *Z_i_* ∼ *Z* for every i∈N. Suppose that *u* ≥ 0 is the number of epithelial cells that turn into fibre cells with hexagonal intersections given by *ρ* and *w* dimensions. If *u* = 0, then we have a standard branching process dynamic and the corresponding CV is exactly as described (E 6). If *u* > 0, then we have a process with emigration, which corresponds to our model. In both cases we start with the total number of ‘offspring cells’, given by

$$O=\overset{X}{\underset{i=1}{\sum }}Z_{i}.$$

If we denote by Δ*X* the increment in the number of epithelial cells, then the basic equation is

E 8 ΔX+u=O−X.

Consider the subcase *u* = 0, first. Using (E 3) and (E 4), we obtain

E 9 $$\begin{array}{rl} E(X+\Delta X) & =E(X)E(Z)\\ \text{and}\;\text{Var(}X+\Delta X\text{)} & =\text{Var(}Z\text{)}E(X)+{\left[E(Z)\right]}^{2}\text{Var}(X). \end{array}\}$$

Therefore, the single cycle CV, under *u* = 0 assumption, is

E 10 $$\text{CV}=\sqrt{\frac{\text{Var}(Z)}{E(X){\left[E(Z)\right]}^{2}}+\frac{\text{Var}(X)}{{\left[E(X)\right]}^{2}}}.$$

For a process of this kind having *t* steps (*t* cell-cycles here) and starting with $X_{0}\equiv N$ cells, we obtain

E 11 $$E(X_{t})=N\cdot E{\left(Z\right)}^{t}$$

and

E 12 $$\text{Var(}X_{t}\text{)}=N\text{Var}(Z){\left[E(Z)\right]}^{t-1}\cdot \frac{{\left[E(Z)\right]}^{t}-1}{E(Z)-1}.$$

This leads to the standard branching process CV at time *t* formula, i.e.

E 13 $$\text{C}{\text{V}}_{t}=\sqrt{\frac{\text{Var}(Z)}{E(Z)[E(Z)-1]}}\cdot \frac{1}{\sqrt{N}}\sqrt{1-\frac{1}{{\left[E(Z)\right]}^{t}}}.$$

Let us compare this to the case *u* > 0, which corresponds to our model. The geometry of the sphere leads to

E 14 2ρwu=a[(O−u)−X],

i.e. to

E 15 $$u=\frac{a}{2\rho w+a}(O-X).$$

If we denote

E 16 $$\lambda :=\frac{2\rho w}{2\rho w+a}\in \langle 0,1\rangle ,$$

then

E 17 X+ΔX=λO+(1−λ)X.

Introducing the quantity H:=λE(Z)+1−λ<E(Z), we obtain

E 18 $$\begin{array}{rl} E(X+\Delta X) & =E(X)H\\ \text{and}\;\text{Var}(X+\Delta X) & =H^{2}\text{Var}(X)+\lambda^{2}\text{Var(}Z\text{)}E(X). \end{array}\}$$

The corresponding (single cycle) CV is

E 19 $$\text{C}{\text{V}}^{\lambda }=\sqrt{\frac{\lambda^{2}\text{Var}(Z)}{E(X)H^{2}}+\frac{\text{Var}(X)}{{\left[E(X)\right]}^{2}}}.$$

In order to compare (E 10) and (E 19), i.e. standard single-cycle CV with ‘emigration-corrected’ CV, observe the factor

E 20 $$\frac{\lambda /H}{1/E(Z)}=\frac{1}{1+((1-\lambda )/\lambda )\cdot 1/E(Z)}=\frac{2\rho w(1+p)}{2\rho w(1+p)+a}<1;$$

in the prenatal period it ranges somewhere between 1/2 and 1/3. Typical behaviour with time is that 2*ρw* increases moderately, *p* decreases significantly and *a* increases rather sharply. For example, for 2*ρw* = 40, *p* = 0.1 and *a* = 200, the factor in (E 20) would be 11/61 ≈ 1/6.

For stable parameters through *t* cycles we would obtain

E 21 $$E(X_{t}^{\lambda })=N\cdot H^{t}$$

and

E 22 $$\text{ Var}(X_{t}^{\lambda })=N\text{Var}(Z)\lambda^{2}H^{t-1}\cdot \frac{H^{t}-1}{H-1}.$$

Hence, we obtain

E 23 $${\text{CV}}_{t}^{\lambda }=\lambda \sqrt{\frac{\text{Var}(Z)}{H[H-1]}}\cdot \frac{1}{\sqrt{N}}\sqrt{1-\frac{1}{H^{t}}}.$$

It follows that for *λ* ‘big’ (say *λ* > 1/2 ; which is atypical for our process) the ratio

$$\frac{{\text{CV}}_{t}^{\lambda }}{\text{C}{\text{V}}_{t}}\approx \lambda .$$

However, for a mouse older than a month, *E*(*Z*) goes typically close to 1, while *λ* would roughly change from 1/5 to 1/7. For *E*(*Z*) ≈ 1, we have

E 24 $$\frac{{\text{CV}}_{t}^{\lambda }}{\text{C}{\text{V}}_{t}}\approx \sqrt{\lambda }.$$

This principle essentially does not change even within our full model. However, owing to a four zone structure, the formulae are more difficult to follow. In the following section, we provide the details.

*Four Zone Structure*. We follow our complete model description as given in appendix A. For *i* = 1, 2, 3, 4 we denote by Δ*X_i_* the increment at time *t* in zone *i*. The equations of our model that provide the basic dynamics between the zones are (see appendix A)

E 25 $$\begin{array}{rl} \Delta X_{1} & =x,\\ \Delta X_{2} & =(O_{2}-X_{2})-(x+y),\\ \Delta X_{3} & =(O_{3}-X_{3})-z+y\\ \text{and}\;\Delta X_{4} & =z-u, \end{array}\}$$

where *O*_2_ are offspring cells developed in PGZ with branching distribution

$$Z∼\left(\begin{array}{ccc} 0 & 1 & 2\\ 0 & 1-q & q \end{array}\right),$$

and *O*_3_ are offspring cells developed in GZ with branching distribution

$$W∼\left(\begin{array}{ccc} 0 & 1 & 2\\ 0 & 1-r & r \end{array}\right),$$

and *u* is the number of cells that leave epithelium. This leads to a basic equation of the form

E 26 $$\Delta X+u=(O_{2}-X_{2})+(O_{3}-X_{3}),$$

where *X *= *X*_1_ + *X*_2_ + *X*_3_ + *X*_4_. We consider the case *u* > 0 here. Denote by *G* the deterministic function defined by

$$G(t)=\overset{4}{\underset{i=1}{\sum }}\frac{\eta_{i}(t)}{a_{i}(t)};$$

it follows that

E 27 X(t)=G(t)A(t).

As ΔA=2ρ(t)w(t)⋅u, (E 26) leads to

E 28 $$u+G(t+1)2\rho (t)w(t)\cdot u+A(t)[G(t+1)-G(t)]=(O_{2}-X_{2})+(O_{3}-X_{3}).$$

Let us introduce deterministic functions *λ *= *λ*(*t*) and *µ *= *µ*(*t*) by

E 29 $$\lambda (t)=\frac{2\rho (t)w(t)G(t+1)}{1+2\rho (t)w(t)G(t+1)}$$

and

E 30 $$\mu (t)=\frac{G(t+1)}{G(t)}.$$

It is now not difficult to check that (E 28) has a form analogous to (E 17), i.e.

E 31 $$X+\Delta X=\lambda (O_{2}-X_{2})+\lambda (O_{3}-X_{3})+\lambda X+\mu (1-\lambda )X.$$

Recall that, for *i *= 1, 2, 3, 4, *X_i_*(*t*) = *v_i_*(*t*)*X*(*t*), where *v_i _*= *v_i_*(*t*) is defined by

$$\nu_{i}(t):=\frac{\eta_{i}(t)/a_{i}(t)}{G(t)}.$$

As, for $i=2,3,E(O_{i})-E(X_{i})=E(X_{i})(1+p_{i}-1)=E(X_{i})p_{i}$, where *p*_2_ = *q* and *p*_3_ = *r*, we obtain

$$\begin{array}{rl} E(X+\Delta X) & =\lambda (q\nu_{2}+r\nu_{3}+1)E(X)+\mu (1-\lambda )E(X)\\ & =E(X)[\lambda (q\nu_{2}+r\nu_{3}+1)+(1-\lambda )\mu ]. \end{array}$$

In order to calculate *E*((*X*+Δ*X*)^2^), we need formulae of the form (E 7). Observe that *X_i_*(*t*) = *v_i_*(*t*)*X*(*t*) in practical applications (i.e. there is an integer-valued function *h*). However, because *X* is of the order 10^4^, we shall neglect (as we described in [5]) the error smaller than 10^−4^ and use the formula that

$$E(X\cdot \lfloor \nu_{i}(t)X(t)\rfloor )=E(\nu_{i}(t)\cdot X^{2})=\nu_{i}(t)E(X^{2}).$$

Therefore, using (E 7) and analogous formulae, we obtain

$$\begin{array}{rl} E(O_{2}\cdot O_{3}) & =E(Z)E(W)\frac{\nu_{3}}{\nu_{2}}E(X_{2}^{2})=\nu_{2}\nu_{3}E(Z)E(W)E(X^{2}),\\ E(O_{2}\cdot O_{3}) & =\frac{\nu_{3}}{\nu_{2}}E(O_{2}X_{2})=\frac{\nu_{3}}{\nu_{2}}E(Z)E(X_{2}^{2})=\nu_{2}\nu_{3}E(Z)E(X^{2}),\\ E(O_{3}\cdot X_{2}) & =\frac{\nu_{2}}{\nu_{3}}E(O_{3}X_{3})=\frac{\nu_{2}}{\nu_{3}}E(W)E(X_{3}^{2})=\nu_{2}\nu_{3}E(W)E(X^{2}),\\ E(O_{2}\cdot X) & =\frac{1}{\nu_{2}}E(O_{2}X_{2})=\frac{1}{\nu_{2}}E(Z)E(X_{2}^{2})=\nu_{2}E(Z)E(X^{2})\\ \text{and}\;E(O_{3}\cdot X) & =\frac{1}{\nu_{3}}E(O_{3}X_{3})=\frac{1}{\nu_{3}}E(W)E(X_{3}^{2})=\nu_{3}E(W)E(X^{2}). \end{array}$$

This enables us to calculate $E({\left(X+\Delta X\right)}^{2})$ and Var(X+ΔX). Using the notation $\tilde{H}:=\lambda (q\nu_{2}+r\nu_{3}+1)+(1-\lambda )\mu$, we obtain

E 33 $$E({\left(X+\Delta X\right)}^{2})=\lambda^{2}E(X)(\nu_{2}\text{Var}(Z)+\nu_{3}\text{Var}(W))+{\tilde{H}}^{2}E(X^{2}).$$

This leads to the formula for $\text{C}{\text{V}}^{\lambda ,\mu }$ in this case

E 34 $$\text{C}{\text{V}}^{\lambda ,\mu }=\sqrt{\frac{\lambda^{2}(\nu_{2}\text{Var}(Z)+\nu_{3}\text{Var}(W))}{E(X)\cdot {\tilde{H}}^{2}}+\frac{\text{Var}(X)}{{\left[E(X)\right]}^{2}}};$$

which is analogous to (E 19).

## Appendix F. Cluster size estimates and transit time

We provide elements of the spatial movement of individual cells and their progeny through the epithelium. The analysis is simplified on several accounts and is a study of averages only. Nevertheless, it gives us the essence of the laws that characterize such movements through the epithelium.

We consider the epithelium of the mouse lens as a half-sphere of radius *R*. During early stages, the entire lens epithelium acts as a single GZ, and is covered by a single layer of cells. On average each cell covers the surface area *a* (given in square micrometres). Furthermore, we assume that the epithelium is covered by L∈N concentric latitudinal layers (lines of latitude on the surface of the Earth are a good analogy), where every layer has the same width, equal to $\sqrt{a}$. Hence,

F 1 $$L=\frac{1}{\sqrt{a}}\cdot \frac{R\pi }{2}.$$

As the zonal structure of the epithelium does not have too much of an impact on (F1) until P7, we obtain the following table, based on (F1):

$$\begin{array}{cccc} & R(\text{μ}m) & a(\mu {\text{m}}^{2}) & L\\ E14 & 230 & 30 & 65\\ E16 & 350 & 36 & 91\\ E18 & 420 & 42.2 & 101\\ P1 & 462 & 46 & 107\\ P7 & 640 & 65 & 124. \end{array}$$

Between P7 and P21, the zonal structure of the lens develops, where PGZ and GZ are zones with active proliferation. By P21, the number of layers in both zones stabilizes at around 50 layers.

The second set of simplifications covers the description of the spatial movement of a cell and its daughter cells. Suppose that we observe a single epithelial cell within a zone with proliferation rate p∈0,1. We are interested in two quantities, the position of the cell and the total number of its daughter cells, i.e. a stochastic process (*Z_t_*, *Y_t_*). The interpretation of $Z_{t}\in N$ is the number of layers above (i.e. ‘to the north of’) the cell at time $t\in N_{0}$. Suppose that we have m∈N layers above our cell. We assume that the probability of each layer ‘pushing’ our cell for a single layer ‘southward’ is *p*. According to our general model, cells within the epithelium act independently, which implies that layers act independently (because they are disjoint subfamilies of an independent family of random variables). The consequence is that the ‘total push’ of *m* layers acts as a binomial random variable *B*(*m*, *p*). Hence, the probability that at time *t* + 1 the cell will be in a position *m *+ *k*, 0 ≤ *k *≤ *m* is

$$\left(\begin{array}{c} m\\ k \end{array}\right)p^{k}{\left(1-p\right)}^{m-k}.$$

It follows that the average movement ‘southward’ in a single time interval, starting from a position *m*, is

F 2 mp.

As the cell started at *m*, at *t* + 1 it will be, on average, at

F 3 m+mp=m(1+p).

Owing to the independence assumption, the process (*Z_t_*) has a (discrete time and discrete state space) Markov property. It follows then (using (F 3)) that, if the cell was initially positioned at $Z_{0}\equiv m$, and proliferation rates in the next *N* time intervals were *p*_1_, *p*_2_, … ,*p_N_*, then, on average, the position of the cell as time *N* is

F 4 $$m(1+p_{1})(1+p_{2})\cdot \ldots \cdot (1+p_{N})=m\overset{N}{\underset{k=1}{\prod }}(1+p_{k}).$$

The interpretation of $Y_{t}\in N\cup \{0\}=N_{0}$ is the total number of daughter cells at time *t*. Hence $Y_{0}\equiv 1$. It follows that (*Y_t_*) is a standard branching process with the branching mechanism distribution at *t* = *k* given by

F 5 $$\left(\begin{array}{ccc} 0 & 1 & 2\\ 0 & 1-p_{k} & p_{k} \end{array}\right).$$

The well-known formula for the average value is

F 6 $$E(Y_{N})=\overset{N}{\underset{k=1}{\prod }}(1+p_{k}).$$

We are interested in a ‘cluster of cells’ produced by the original cell. By ‘cluster’ we mean all daughter cells that remain in ‘close proximity’ throughout their movement within the epithelium. Therefore, the worst case scenario is

F 7 cluster size=E(Y)

and we shall use (F 7) as the ‘cluster size’ (it is actually an average upper bound on the ‘cluster size’).

Suppose that our cell starts at some level *L*_0_ and, on average, after *N* time intervals (for us a single time interval corresponds to a single cell cycle; after E18 this is a period of 24 h) our cluster reaches some level *L*_∞_, where *L*_0_ < *L*_∞_. Then the cluster size does not depend on proliferation rates; it depends only on the ratio of the levels, i.e. we have

F 8 $$E(Y_{N})=\frac{L_{\infty }}{L_{0}}.$$

The result follows easily from our formulae

$$E(Y_{N})=\overset{N}{\underset{k=1}{\prod }}(1+p_{k})=\frac{L_{0}}{L_{0}}\overset{N}{\underset{k=1}{\prod }}(1+p_{k})=\frac{\text{Avg. Pos.}}{L_{0}}=\frac{L_{\infty }}{L_{0}}.$$

This result is perhaps unexpected, but the intuition behind it is that higher proliferation rates will increase the cluster size faster, but at the same time the cluster will travel ‘southward’ at a higher speed.

Our primary interest is in the cluster size that will enter the fibre compartment, i.e. in the case *L*_∞_ = *L*. Because for most of a mouse life *L* is generally around 100 (with a high probability the initial position of interest is going to be *L*_0_ = 2, *L*_0_ = 3).

Another interesting consequence of our computations is that, although cluster size is not affected by the changes in proliferation rate, travel time is. Let us denote by *N*_∞_ the time (number of cycles, later number of days) required for the average position to reach the equator. It follows that *N*_∞_ satisfies the equation

F 9 $$\overset{N_{\infty }}{\underset{k=1}{\prod }}(1+p_{k})=\frac{L}{L_{0}}.$$

At a later stage, when the proliferation rate stabilizes, we obtain the formula

F 10 $$N_{\infty }=\frac{\text{ln}L/L_{0}}{\text{ln(}1+p\text{)}}.$$

Let us illustrate the importance of this result on a particular example. Suppose that a CZ cell enters PGZ on the level *L*_0_ = 2, and then travels, say *L* = 100, to the equator. If that occurs in a three-month-old mouse, average *p* will be 4%, i.e.

$$N_{\infty }=\frac{\ln50}{\ln(1.04)}\approx 100\;\text{days.}$$

Hence, we can expect a cluster of 50 cells to have entered the fibre compartment by the time the mouse is about seven months old. If, on the other hand, the migration process commenced in a six-month-old mouse, the average *p* will be 1%, i.e.

$$N_{\infty }=\frac{\ln50}{\ln(1.01)}\approx 395\;\text{days.}$$

Thus, the mouse would be over a year and a half old by the time the cell cluster arrived at the lens equator.

Among the most detailed information, we possess are data on proliferation rates. This enables us to calculate exactly the key factor

$$\overset{N}{\underset{k=1}{\prod }}(1+p_{k}).$$

This factor appears in most of our formulae. For example, if we start at *t* = 0 which corresponds to E14.5, then we obtain the following table.

$$\begin{array}{ccc} \text{age} & N & \overset{N}{\underset{k=1}{\prod }}(1+p_{k})\\ E14.5 & 1 & 1.82\\ E16 & 4 & 8.1\\ E18 & 7 & 21.54\\ P1 & 10 & 41.07 \end{array}$$

This implies that every cell with *L*_0_ ≥ 3 would have enough average time to leave epithelium by P1 (of course, that does not mean that it will actually do so; the outcomes of this process are stochastic).

If the cell travels within the PGZ only, the formulae above apply directly. However, when the cell (or its cluster) moves into GZ, we have to account for the differing proliferation rates of the two zones. Let us denote the rate in PGZ by *q* and the rate in GZ by *r*. We always have 0 < *q *< *r *< 1. Suppose that PGZ has the total of :*L*_PGZ_ layers and that our cluster is positioned so that there are *m* layers within GZ above it. Then, the probability that at the same time PGZ layers produce a ‘push’ of size *j* and GZ layers a push of size *k* is

$$\left(\begin{array}{c} L_{\mathrm{PGZ}}\\ j \end{array}\right)q^{j}{\left(1-q\right)}^{L_{\mathrm{PGZ}}-j}\cdot \left(\begin{array}{c} m\\ k \end{array}\right)r^{k}{\left(1-r\right)}^{m-k}.$$

Hence the average movement ‘southward’ is

$$qL_{\mathrm{PGZ}}+rm,$$

and the average position at the end of the cycle is

F 11 $$L_{\mathrm{PGZ}}+m+qL_{\mathrm{PGZ}}+rm=(L_{\mathrm{PGZ}}+m)(1+r-\Delta ),$$

where ‘the correction factor’ Δ is given by

F 12 $$\Delta =\frac{L_{\mathrm{PGZ}}(r-q)}{L_{\mathrm{PGZ}}+m}.$$

Observe that the factor (1 + *r *− Δ) is not going to cancel with a corresponding factor in (E 6) (which is of the form (1 + *r*)). In other words, formula (E 8) will be corrected via a finite number of factors of the form

F 13 $$\frac{1+r}{1+r-\Delta }.$$

We can estimate Δ based on our data. Using midpoint estimate for *m* ≈ 1/2 *L*_PGZ_, we obtain Δ ≈ 2/3(*r *− *q*). The ratio of *r* and *q* changes from say, a one-month-old mouse, when *q* = 1/3*r* to a 3+ year-old mouse, when *q* = 1/5*r*. Hence, for a ≈one-month-old mouse (*r* = 0.06) the factor in (F 13) is approximately

$$\frac{1+r}{1+(5/9)r}\approx 1.0258,$$

while for a ≈3-year-old mouse *r* = 0.01 it is

$$\frac{1+r}{1+(7/15)r}\approx 1.00531.$$

The factor 1.00531 is very close to 1 and over a period of a few weeks will not have a significant effect on the cluster size. However, in the unlikely case (even under laboratory conditions) that a 3-year-old mouse lives to be 4 years old, the effect becomes significant. More precisely,

$$1.00531^{365}\approx 6.91.$$

This suggests that an expected cluster of, say size 100, may turn out to be of the size close to 700, which would cover most of the lens equator.
