# Supplementary material for: A full lifespan model of vertebrate lens growth
Source: R Soc Open Sci. 2017 Jan 18;4(1):160695. doi: 10.1098/rsos.160695 (PMC5319337; doi:10.1098/rsos.160695)
Supplement: MATLAB code for running the full lifespan lens growth model [file rsos160695supp1.docx]

**MATLAB code required to run the full lifespan lens growth model.**

Main program:

function var=wholelife_2()

drawpom=mark7_e_p_sapoc_2();

nfirst=length(drawpom(1,:));

var(1,1:nfirst)=drawpom(1,:);

var(2,1:nfirst)=drawpom(2,:);

var(3,1:nfirst)=drawpom(3,:);

var(4,1:nfirst)=drawpom(4,:);

var(5,1:nfirst)=drawpom(5,:);

var(6,1:nfirst)=drawpom(6,:);

var(7,1:(nfirst-1))=drawpom(7,1:(nfirst-1));

var(8,1:(nfirst-1))=drawpom(8,1:(nfirst-1));

var(9,1:(nfirst-1))=drawpom(9,1:(nfirst-1));

var(10,1:(nfirst-1))=drawpom(10,1:(nfirst-1));

var(11,1:(nfirst-1))=drawpom(11,1:(nfirst-1));

var(12,1)=0;

var(12,2:nfirst)=drawpom(12,1:(nfirst-1));

var(13,1)=0;

var(13,2:nfirst)=drawpom(13,1:(nfirst-1));

var(14,1)=200*3/20;

var(14,2:nfirst)=drawpom(14,1:(nfirst-1));

var(15,1)=0;

var(15,2:nfirst)=drawpom(15,1:(nfirst-1));

var(16,1)=0;

var(16,2:nfirst)=drawpom(16,1:(nfirst-1));

var(17,1)=0;

var(17,2:nfirst)=drawpom(17,1:(nfirst-1));

var(18,1)=1;

var(18,2:nfirst)=drawpom(18,1:(nfirst-1));

var(19,1)=0;

var(19,2:nfirst)=drawpom(19,1:(nfirst-1));

beta_5=drawpom(20,5);

beta_6=drawpom(20,6);

beta_7=drawpom(20,7);

beta_8=drawpom(20,8);

N1=drawpom(20,1);

N2=drawpom(20,2);

N3=drawpom(20,3);

N4=drawpom(20,4);

draw_dip=allfhases_2(0,0,0,0,60,90,540,720,10,2,200,0,0,1,0,0.32,0.33,0.26,0.09,0,0,0,0,0,0,-0.05/7,0.05/7,0.1/7,0.2/7,-0.34/7,0.04/7,0.22/14,0.13/14,-0.35/14,0,0,0,0,0,0,0,0,0,0,0,0,0,0,0,0,0,beta_5,beta_6,beta_7,beta_8,0,0,0,0,1/70,1/70,1/70,1/140,1/20,1/35,3/140,1/140,3/280,3/280,1/140,1/560,1/200,3/400,7/800,11/2400,0,1/900,1/900,1/900,1/2700,1/5400,1/5400,1/5400,1/7200,1/7200,1/9600,1/7200,N1,N2,N3,N4,0,1,0,0,1,0,0,1,0,0,1,0,0,1,0,0,1,0,0,1,0,0,1,0,0,1,0,0,1,0,0,0.88,0.12,0,0.94,0.06,0,0.98,0.02,0,0.995,0.005,0,0.997,0.003,0,0.998,0.002,0,0.74,0.26,0,0.82,0.18,0,0.84,0.16,0,0.92,0.08,0,0.94,0.06,0,0.985,0.015,0,0.988,0.012,0,0.99,0.01,0,0,0,0,0,0,0.0003,0.00025);

nsecond=length(draw_dip(1,:));

var(1,(nfirst+1):(nfirst+nsecond-1))=draw_dip(1,2:nsecond);

var(2,(nfirst+1):(nfirst+nsecond-1))=draw_dip(2,2:nsecond);

var(3,(nfirst+1):(nfirst+nsecond-1))=draw_dip(3,2:nsecond);

var(4,(nfirst+1):(nfirst+nsecond-1))=draw_dip(4,2:nsecond);

var(5,(nfirst+1):(nfirst+nsecond-1))=draw_dip(5,2:nsecond);

var(6,(nfirst+1):(nfirst+nsecond-1))=draw_dip(6,2:nsecond);

var(7,nfirst:(nfirst+nsecond-2))=draw_dip(7,1:(nsecond-1));

var(8,nfirst:(nfirst+nsecond-2))=draw_dip(8,1:(nsecond-1));

var(9,nfirst:(nfirst+nsecond-2))=draw_dip(9,1:(nsecond-1));

var(10,nfirst:(nfirst+nsecond-2))=draw_dip(10,1:(nsecond-1));

var(11,nfirst:(nfirst+nsecond-2))=draw_dip(11,1:(nsecond-1));

var(12,(nfirst+1):(nfirst+nsecond-1))=draw_dip(12,1:(nsecond-1));

var(13,(nfirst+1):(nfirst+nsecond-1))=draw_dip(13,1:(nsecond-1));

var(14,(nfirst+1):(nfirst+nsecond-1))=draw_dip(14,1:(nsecond-1));

var(15,(nfirst+1):(nfirst+nsecond-1))=draw_dip(15,1:(nsecond-1));

var(16,(nfirst+1):(nfirst+nsecond-1))=draw_dip(16,1:(nsecond-1));

var(17,(nfirst+1):(nfirst+nsecond-1))=draw_dip(17,1:(nsecond-1));

var(18,(nfirst+1):(nfirst+nsecond-1))=draw_dip(18,1:(nsecond-1));

var(19,(nfirst+1):(nfirst+nsecond-1))=draw_dip(19,1:(nsecond-1));

end

Subprogram mark7_e_p_sapoc_2.m:

function var=mark7_e_p_sapoc_2()

beta_1=3/20;

beta_2=3/20;

beta_3=3/20;

beta_4=3/20;

N1=0;

N2=0;

N3=11000;

N4=0;

draw=mark7_e14_e21_sapoc(N1,N2,N3,N4,beta_1,beta_2,beta_3,beta_4);

var(1,1)=draw(1,1);

var(1,2)=draw(1,3);

var(1,3)=draw(1,5);

var(1,4)=(draw(1,6)+draw(1,7))/2;

var(1,5)=draw(1,8);

var(1,6:8)=draw(1,9:11);

var(2,1)=draw(2,1);

var(2,2)=draw(2,3);

var(2,3)=draw(2,5);

var(2,4)=(draw(2,6)+draw(2,7))/2;

var(2,5)=draw(2,8);

var(2,6:8)=draw(2,9:11);

var(3,1)=draw(3,1);

var(3,2)=draw(3,3);

var(3,3)=draw(3,5);

var(3,4)=(draw(3,6)+draw(3,7))/2;

var(3,5)=draw(3,8);

var(3,6:8)=draw(3,9:11);

var(3,1)=draw(3,1);

var(3,2)=draw(3,3);

var(3,3)=draw(3,5);

var(3,4)=(draw(3,6)+draw(3,7))/2;

var(3,5)=draw(3,8);

var(3,6:8)=draw(3,9:11);

var(4,1)=draw(4,1);

var(4,2)=draw(4,3);

var(4,3)=draw(4,5);

var(4,4)=(draw(4,6)+draw(4,7))/2;

var(4,5)=draw(4,8);

var(4,6:8)=draw(4,9:11);

var(5,1)=draw(5,1);

var(5,2)=draw(5,3);

var(5,3)=draw(5,5);

var(5,4)=(draw(5,6)+draw(5,7))/2;

var(5,5)=draw(5,8);

var(5,6:8)=draw(5,9:11);

var(6,1)=draw(6,1);

var(6,2)=draw(6,3);

var(6,3)=draw(6,5);

var(6,4)=(draw(6,6)+draw(6,7))/2;

var(6,5)=draw(6,8);

var(6,6:8)=draw(6,9:11);

var(7,1)=draw(7,1)+draw(7,2);

var(7,2)=draw(7,3)+draw(7,4);

var(7,3)=draw(7,5)+draw(7,6)/2;

var(7,4)=draw(7,6)/2+draw(7,7);

var(7,5)=draw(7,8);

var(7,6:7)=draw(7,9:10);

var(8,1)=draw(8,1)+draw(8,2);

var(8,2)=draw(8,3)+draw(8,4);

var(8,3)=draw(8,5)+draw(8,6)/2;

var(8,4)=draw(8,6)/2+draw(8,7);

var(8,5)=draw(8,8);

var(8,6:7)=draw(8,9:10);

var(9,1)=draw(9,1)+draw(9,2);

var(9,2)=draw(9,3)+draw(9,4);

var(9,3)=draw(9,5)+draw(9,6)/2;

var(9,4)=draw(9,6)/2+draw(9,7);

var(9,5)=draw(9,8);

var(9,6:7)=draw(9,9:10);

var(10,1)=draw(10,1)+draw(10,2);

var(10,2)=draw(10,3)+draw(10,4);

var(10,3)=draw(10,5)+draw(10,6)/2;

var(10,4)=draw(10,6)/2+draw(10,7);

var(10,5)=draw(10,8);

var(10,6:7)=draw(10,9:10);

var(11,1)=draw(11,1);

var(11,2)=draw(11,3);

var(11,3)=draw(11,5);

var(11,4)=(draw(11,6)+draw(11,7))/2;

var(11,5)=draw(11,8);

var(11,6:7)=draw(11,9:10);

var(12,1:7)=0;

var(13,1:7)=0;

var(14,1)=draw(14,2);

var(14,2)=draw(14,4);

var(14,3)=(draw(14,5)+draw(14,6))/2;

var(14,4)=draw(14,7);

var(14,5)=draw(14,8);

var(14,6:7)=draw(14,9:10);

var(15,1:7)=0;

var(16,1:7)=0;

var(17,1:7)=0;

var(18,1:7)=1;

var(19,1:7)=0;

beta_1=44/200;

beta_2=44/200;

beta_3=44/200;

beta_4=44/200;

draw2=mark7_p_sapoc_2(0,0,draw(1,11),0,beta_1,beta_2,beta_3,beta_4);

var(1,9:36)=draw2(1,2:29);

var(2,9:36)=draw2(2,2:29);

var(3,9:36)=draw2(3,2:29);

var(4,9:36)=draw2(4,2:29);

var(5,9:36)=draw2(5,2:29);

var(6,9:36)=draw2(6,2:29);

var(7,8:35)=draw2(7,1:28);

var(8,8:35)=draw2(8,1:28);

var(9,8:35)=draw2(9,1:28);

var(10,8:35)=draw2(10,1:28);

var(11,8:35)=draw2(11,1:28);

var(12,8:35)=draw2(12,1:28);

var(13,8:35)=draw2(13,1:28);

var(14,8:35)=draw2(14,1:28);

var(15,8:35)=draw2(15,1:28);

var(16,8:35)=draw2(16,1:28);

var(17,8:35)=draw2(17,1:28);

var(18,8:35)=draw2(18,1:28);

var(19,8:35)=draw2(19,1:28);

var(20,1)=draw2(20,1);

var(20,2)=draw2(20,2);

var(20,3)=draw2(20,3);

var(20,4)=draw2(20,4);

var(20,5)=draw2(20,5);

var(20,6)=draw2(20,6);

var(20,7)=draw2(20,7);

var(20,8)=draw2(20,8);

end

Subprogram mark7_e14_e21_sapoc.m:

function var=mark7_e14_e21_sapoc(N1,N2,N3,N4,beta_1,beta_2,beta_3,beta_4)

n1=4;

r12poc=0.82;

draw1=mark7e14_e21_stlouis(n1,6.3,1.2,200,0,0,1,0,0,0,0,0,beta_1,beta_2,beta_3,beta_4,1/280,1/280,2/280,1/280,N1,N2,N3,N4,0,0,0,0,0,0,0,1-r12poc,r12poc,0);

n2=3;

beta_3=n1*1/140+beta_3;

N3=draw1(1,(n1+1));

r12poc=0.48;

draw2=mark7e14_e21_stlouis2(n2,6.3,1.2,200,0,0,1,0,0,0,0,0,3/20,3/20,beta_3,3/20,1/280,1/280,3/280,1/280,0,0,N3,0,0,0,0,0,0,0,0,1-r12poc,r12poc,0);

beta_3=beta_3+n2*3/280;

n3=3;

N3=draw2(1,(n2+1));

r12poc=0.25;

draw3=mark7e14_e21_stlouis3(n3,6.3,1.2,200,0,0,1,0,0,0,0,0,3/20,3/20,beta_3,3/20,1/280,1/280,1/2000,1/280,0,0,N3,0,0,0,0,0,0,0,0,1-r12poc,r12poc,0);

var(1,1:(n1+1))=draw1(1,:);

var(1,(n1+2):(n1+n2+1))=draw2(1,2:(n2+1));

var(1,(n1+n2+2):(n1+n2+n3+1))=draw3(1,2:(n3+1));

var(2,1:(n1+1))=draw1(2,:);

var(2,(n1+2):(n1+n2+1))=draw2(2,2:(n2+1));

var(2,(n1+n2+2):(n1+n2+n3+1))=draw3(2,2:(n3+1));

var(3,1:(n1+1))=draw1(3,:);

var(3,(n1+2):(n1+n2+1))=draw2(3,2:(n2+1));

var(3,(n1+n2+2):(n1+n2+n3+1))=draw3(3,2:(n3+1));

var(4,1:(n1+1))=draw1(4,:);

var(4,(n1+2):(n1+n2+1))=draw2(4,2:(n2+1));

var(4,(n1+n2+2):(n1+n2+n3+1))=draw3(4,2:(n3+1));

var(5,1:(n1+1))=draw1(5,:);

var(5,(n1+2):(n1+n2+1))=draw2(5,2:(n2+1));

var(5,(n1+n2+2):(n1+n2+n3+1))=draw3(5,2:(n3+1));

var(6,1:(n1+1))=draw1(6,:);

var(6,(n1+2):(n1+n2+1))=draw2(6,2:(n2+1));

var(6,(n1+n2+2):(n1+n2+n3+1))=draw3(6,2:(n3+1));

var(7,1:n1)=draw1(7,1:n1);

var(7,(n1+1):(n1+n2))=draw2(7,1:n2);

var(7,(n1+n2+1):(n1+n2+n3))=draw3(7,1:n3);

var(8,1:n1)=draw1(8,1:n1);

var(8,(n1+1):(n1+n2))=draw2(8,1:n2);

var(8,(n1+n2+1):(n1+n2+n3))=draw3(8,1:n3);

var(9,1:n1)=draw1(9,1:n1);

var(9,(n1+1):(n1+n2))=draw2(9,1:n2);

var(9,(n1+n2+1):(n1+n2+n3))=draw3(9,1:n3);

var(10,1:n1)=draw1(10,1:n1);

var(10,(n1+1):(n1+n2))=draw2(10,1:n2);

var(10,(n1+n2+1):(n1+n2+n3))=draw3(10,1:n3);

var(11,1:n1)=draw1(11,1:n1);

var(11,(n1+1):(n1+n2))=draw2(11,1:n2);

var(11,(n1+n2+1):(n1+n2+n3))=draw3(11,1:n3);

var(12,1:n1)=draw1(12,1:n1);

var(12,(n1+1):(n1+n2))=draw2(12,1:n2);

var(12,(n1+n2+1):(n1+n2+n3))=draw3(12,1:n3);

var(13,1:n1)=draw1(13,1:n1);

var(13,(n1+1):(n1+n2))=draw2(13,1:n2);

var(13,(n1+n2+1):(n1+n2+n3))=draw3(13,1:n3);

var(14,1:n1)=draw1(14,1:n1);

var(14,(n1+1):(n1+n2))=draw2(14,1:n2);

var(14,(n1+n2+1):(n1+n2+n3))=draw3(14,1:n3);

var(15,1:n1)=draw1(15,1:n1);

var(15,(n1+1):(n1+n2))=draw2(15,1:n2);

var(15,(n1+n2+1):(n1+n2+n3))=draw3(15,1:n3);

var(16,1:n1)=draw1(16,1:n1);

var(16,(n1+1):(n1+n2))=draw2(16,1:n2);

var(16,(n1+n2+1):(n1+n2+n3))=draw3(16,1:n3);

var(17,1:n1)=draw1(17,1:n1);

var(17,(n1+1):(n1+n2))=draw2(17,1:n2);

var(17,(n1+n2+1):(n1+n2+n3))=draw3(17,1:n3);

var(18,1:n1)=draw1(18,1:n1);

var(18,(n1+1):(n1+n2))=draw2(18,1:n2);

var(18,(n1+n2+1):(n1+n2+n3))=draw3(18,1:n3);

var(19,1:n1)=draw1(19,1:n1);

var(19,(n1+1):(n1+n2))=draw2(19,1:n2);

var(19,(n1+n2+1):(n1+n2+n3))=draw3(19,1:n3);

end

Subprogram mark7e14_e21_stlouis.m:

function var=mark7e14_e21_stlouis(n1,ro,w,a,eta_1,eta_2,eta_3,eta_4,ni_11,ni_12,ni_13,ni_14,beta_1,beta_2,beta_3,beta_4,alfa_11,alfa_12,alfa_13,alfa_14,N1,N2,N3,N4,p10,p11,p12,q10,q11,q12,r10,r11,r12poc,delta1)

X1(1)=N1;

X2(1)=N2;

X3(1)=N3;

X4(1)=N4;

X1pom(1)=X1(1);

X2pom(1)=X2(1);

X3pom(1)=X3(1);

X4pom(1)=X4(1);

X(1)=X1(1)+X2(1)+X3(1)+X4(1);

et(1)=eta_1;

et(2)=eta_2;

et(3)=eta_3;

et(4)=eta_4;

beta(1)=beta_1;

beta(2)=beta_2;

beta(3)=beta_3;

beta(4)=beta_4;

alfa(1)=alfa_11;

alfa(2)=alfa_12;

alfa(3)=alfa_13;

alfa(4)=alfa_14;

l(3)=beta(3);

eta(3)=et(3);

R(1)=sqrt((X3(1)*a*l(3))/(2*pi*eta(3)));

for t=2:(n1+1)

r12=r12poc*(t==2)+(r12poc-0.09)*(t==3)+(r12poc-0.09-0.09)*(t==4)+(r12poc-0.09-0.09-0.08)*(t==5)+(r12poc-0.09-0.09-0.08-0.08)*(t==6)+(r12poc-0.04-0.06-0.08-0.12-0.08)*(t==7)+(r12poc-0.04-0.06-0.08-0.12-0.08-0.06)*(t==8)+(r12poc-0.04-0.06-0.08-0.12-0.08-0.06-(t-8)*0.02)*(t>8);

r11=1-r12;

rvar(t-1)=r12;

l(1)=alfa(1)*(t-2)+beta(1);

l(2)=alfa(2)*(t-2)+beta(2);

l(3)=alfa(3)*(t-2)+beta(3);

l(4)=alfa(4)*(t-2)+beta(4);

eta(1)=et(1)+(t-1)*ni_11;

eta(2)=et(2)+(t-1)*ni_12;

eta(3)=et(3)+(t-1)*ni_13;

eta(4)=et(4)+(t-1)*ni_14;

apom(1)=a*l(1);

apom(2)=a*l(2);

apom(3)=a*l(3);

apom(4)=a*l(4);

rpom=X(t-1)/(eta(1)/apom(1)+eta(2)/apom(2)+eta(3)/apom(3)+eta(4)/apom(4));

X1(t-1)=floor((eta(1)/apom(1))*rpom);

X2(t-1)=floor((eta(2)/apom(2))*rpom);

X3(t-1)=floor((eta(3)/apom(3))*rpom);

X4(t-1)=floor((eta(4)/apom(4))*rpom);

l(1)=l(1)+alfa(1);

l(2)=l(2)+alfa(2);

l(3)=l(3)+alfa(3);

l(4)=l(4)+alfa(4);

apom(1)=a*l(1);

apom(2)=a*l(2);

apom(3)=a*l(3);

apom(4)=a*l(4);

etapo1(t-1)=eta(1);

etapo2(t-1)=eta(2);

etapo3(t-1)=eta(3);

etapo4(t-1)=eta(4);

apo1(t-1)=a*l(1);

apo2(t-1)=a*l(2);

apo3(t-1)=a*l(3);

apo4(t-1)=a*l(4);

avar1(t-1)=apom(1);

avar2(t-1)=apom(2);

avar4(t-1)=apom(4);

avar3(t-1)=apom(3);

for i=1:4

c(i)=2*eta(i)*ro*w/(a*l(i));

d(i)=alfa(i)/(l(i));

end

O(1)=0;

O(2)=0;

O(3)=0;

for i=1:X1(t-1)

pom1=rand();

if (pom1>p10) && (pom1<=p10+p11)

O(1)=O(1)+1;

end

if (pom1>p10+p11)

O(1)=O(1)+2;

end

end

for i=1:X2(t-1)

pom1=rand();

if (pom1>q10) && (pom1<=q10+q11)

O(2)=O(2)+1;

end

if (pom1>q10+q11)

O(2)=O(2)+2;

end

end

for i=1:X3(t-1)

pom1=rand();

if (pom1>r10) && (pom1<=r10+r11)

O(3)=O(3)+1;

end

if (pom1>r10+r11)

O(3)=O(3)+2;

end

end

rpom=X(t-1)/(eta_1/apom(1)+eta_2/apom(2)+eta_3/apom(3)+eta_4/apom(4));

v=rpom*delta1*(2-delta1)/(2*ro*w);

u=1/(1+c(1)+c(2)+c(3)+c(4))*(d(1)*X1(t-1)+d(2)*X2(t-1)+d(3)*X3(t-1)+d(4)*X4(t-1)+O(1)-X1(t-1)+O(2)-X2(t-1)+O(3)-X3(t-1)-v);

xpom=0;

ypom=0;

zpom=0;

un=floor(u);

xn=floor(xpom);

yn=floor(ypom);

zn=floor(zpom);

X1(t)=O(1)+xn;

X2(t)=O(2)-xn-yn;

X3(t)=O(3)-floor(u);

X4(t)=0;

R(t)=sqrt((X3(t)*a*l(3))/(2*pi*eta(3)));

X(t)=X1(t)+X2(t)+X3(t)+X4(t);

X21(t-1)=xn;

X23(t-1)=yn;

X34(t-1)=zn;

XEbes(t-1)=floor(u+v);

X1pom(t)=X1(t);

X2pom(t)=X2(t);

X3pom(t)=X3(t);

X4pom(t)=X4(t);

end

n=n1;

var(1,:)=X;

var(2,:)=R;

var(3,1:(n+1))=X1pom(1:(n+1));

var(4,1:(n+1))=X2pom(1:(n+1));

var(5,1:(n+1))=X3pom(1:(n+1));

var(6,1:(n+1))=X4pom(1:(n+1));

var(7,1:n)=X21;

var(8,1:n)=X23;

var(9,1:n)=X34;

var(10,1:n)=XEbes;

var(11,1:n)=rvar;

var(12,1:n)=avar1(1:n);

var(13,1:n)=avar2(1:n);

var(14,1:n)=avar3(1:n);

var(15,1:n)=avar4(1:n);

var(16,1:n)=etapo1(1:n);

var(17,1:n)=etapo2(1:n);

var(18,1:n)=etapo3(1:n);

var(19,1:n)=etapo4(1:n);

end

Subprogram mark7e14_e21_stlouis2.m:

function var=mark7e14_e21_stlouis2(n1,ro,w,a,eta_1,eta_2,eta_3,eta_4,ni_11,ni_12,ni_13,ni_14,beta_1,beta_2,beta_3,beta_4,alfa_11,alfa_12,alfa_13,alfa_14,N1,N2,N3,N4,p10,p11,p12,q10,q11,q12,r10,r11,r12poc,delta1)

X1(1)=N1;

X2(1)=N2;

X3(1)=N3;

X4(1)=N4;

X1pom(1)=X1(1);

X2pom(1)=X2(1);

X3pom(1)=X3(1);

X4pom(1)=X4(1);

X(1)=X1(1)+X2(1)+X3(1)+X4(1);

et(1)=eta_1;

et(2)=eta_2;

et(3)=eta_3;

et(4)=eta_4;

beta(1)=beta_1;

beta(2)=beta_2;

beta(3)=beta_3;

beta(4)=beta_4;

alfa(1)=alfa_11;

alfa(2)=alfa_12;

alfa(3)=alfa_13;

alfa(4)=alfa_14;

l(3)=beta(3);

eta(3)=et(3);

R(1)=sqrt((X3(1)*a*l(3))/(2*pi*eta(3)));

for t=2:(n1+1)

r12=r12poc*(t==2)+(r12poc-0.09)*(t==3)+(r12poc-0.09-0.09)*(t==4)+(r12poc-0.09-0.09-0.06)*(t==5)+(r12poc-0.09-0.09-0.08-0.08)*(t==6)+(r12poc-0.04-0.06-0.08-0.12-0.08)*(t==7)+(r12poc-0.04-0.06-0.08-0.12-0.08-0.06)*(t==8)+(r12poc-0.04-0.06-0.08-0.12-0.08-0.06-(t-8)*0.02)*(t>8);

r11=1-r12;

rvar(t-1)=r12;

l(1)=alfa(1)*(t-2)+beta(1);

l(2)=alfa(2)*(t-2)+beta(2);

l(3)=alfa(3)*(t-2)+beta(3);

l(4)=alfa(4)*(t-2)+beta(4);

eta(1)=et(1)+(t-1)*ni_11;

eta(2)=et(2)+(t-1)*ni_12;

eta(3)=et(3)+(t-1)*ni_13;

eta(4)=et(4)+(t-1)*ni_14;

apom(1)=a*l(1);

apom(2)=a*l(2);

apom(3)=a*l(3);

apom(4)=a*l(4);

rpom=X(t-1)/(eta(1)/apom(1)+eta(2)/apom(2)+eta(3)/apom(3)+eta(4)/apom(4));

X1(t-1)=floor((eta(1)/apom(1))*rpom);

X2(t-1)=floor((eta(2)/apom(2))*rpom);

X3(t-1)=floor((eta(3)/apom(3))*rpom);

X4(t-1)=floor((eta(4)/apom(4))*rpom);

l(1)=l(1)+alfa(1);

l(2)=l(2)+alfa(2);

l(3)=l(3)+alfa(3);

l(4)=l(4)+alfa(4);

apom(1)=a*l(1);

apom(2)=a*l(2);

apom(3)=a*l(3);

apom(4)=a*l(4);

etapo1(t-1)=eta(1);

etapo2(t-1)=eta(2);

etapo3(t-1)=eta(3);

etapo4(t-1)=eta(4);

apo1(t-1)=a*l(1);

apo2(t-1)=a*l(2);

apo3(t-1)=a*l(3);

apo4(t-1)=a*l(4);

avar1(t-1)=apom(1);

avar2(t-1)=apom(2);

avar4(t-1)=apom(4);

avar3(t-1)=apom(3);

for i=1:4

c(i)=2*eta(i)*ro*w/(a*l(i));

d(i)=alfa(i)/(l(i));

end

O(1)=0;

O(2)=0;

O(3)=0;

for i=1:X1(t-1)

pom1=rand();

if (pom1>p10) && (pom1<=p10+p11)

O(1)=O(1)+1;

end

if (pom1>p10+p11)

O(1)=O(1)+2;

end

end

for i=1:X2(t-1)

pom1=rand();

if (pom1>q10) && (pom1<=q10+q11)

O(2)=O(2)+1;

end

if (pom1>q10+q11)

O(2)=O(2)+2;

end

end

for i=1:X3(t-1)

pom1=rand();

if (pom1>r10) && (pom1<=r10+r11)

O(3)=O(3)+1;

end

if (pom1>r10+r11)

O(3)=O(3)+2;

end

end

rpom=X(t-1)/(eta_1/apom(1)+eta_2/apom(2)+eta_3/apom(3)+eta_4/apom(4));

v=rpom*delta1*(2-delta1)/(2*ro*w);

u=1/(1+c(1)+c(2)+c(3)+c(4))*(d(1)*X1(t-1)+d(2)*X2(t-1)+d(3)*X3(t-1)+d(4)*X4(t-1)+O(1)-X1(t-1)+O(2)-X2(t-1)+O(3)-X3(t-1)-v);

xpom=0;

ypom=0;

zpom=0;

un=floor(u);

xn=floor(xpom);

yn=floor(ypom);

zn=floor(zpom);

X1(t)=O(1)+xn;

X2(t)=O(2)-xn-yn;

X3(t)=O(3)-floor(u);

X4(t)=0;

R(t)=sqrt((X3(t)*a*l(3))/(2*pi*eta(3)));

X(t)=X1(t)+X2(t)+X3(t)+X4(t);

X21(t-1)=xn;

X23(t-1)=yn;

X34(t-1)=zn;

XEbes(t-1)=floor(u+v);

X1pom(t)=X1(t);

X2pom(t)=X2(t);

X3pom(t)=X3(t);

X4pom(t)=X4(t);

end

n=n1;

var(1,:)=X;

var(2,:)=R;

var(3,1:(n+1))=X1pom(1:(n+1));

var(4,1:(n+1))=X2pom(1:(n+1));

var(5,1:(n+1))=X3pom(1:(n+1));

var(6,1:(n+1))=X4pom(1:(n+1));

var(7,1:n)=X21;

var(8,1:n)=X23;

var(9,1:n)=X34;

var(10,1:n)=XEbes;

var(11,1:n)=rvar;

var(12,1:n)=avar1(1:n);

var(13,1:n)=avar2(1:n);

var(14,1:n)=avar3(1:n);

var(15,1:n)=avar4(1:n);

var(16,1:n)=etapo1(1:n);

var(17,1:n)=etapo2(1:n);

var(18,1:n)=etapo3(1:n);

var(19,1:n)=etapo4(1:n);

end

Subprogram mark7e14_e21_stlouis3.m:

function var=mark7e14_e21_stlouis3(n1,ro,w,a,eta_1,eta_2,eta_3,eta_4,ni_11,ni_12,ni_13,ni_14,beta_1,beta_2,beta_3,beta_4,alfa_11,alfa_12,alfa_13,alfa_14,N1,N2,N3,N4,p10,p11,p12,q10,q11,q12,r10,r11,r12poc,delta1)

X1(1)=N1;

X2(1)=N2;

X3(1)=N3;

X4(1)=N4;

X1pom(1)=X1(1);

X2pom(1)=X2(1);

X3pom(1)=X3(1);

X4pom(1)=X4(1);

X(1)=X1(1)+X2(1)+X3(1)+X4(1);

et(1)=eta_1;

et(2)=eta_2;

et(3)=eta_3;

et(4)=eta_4;

beta(1)=beta_1;

beta(2)=beta_2;

beta(3)=beta_3;

beta(4)=beta_4;

alfa(1)=alfa_11;

alfa(2)=alfa_12;

alfa(3)=alfa_13;

alfa(4)=alfa_14;

l(3)=beta(3);

eta(3)=et(3);

R(1)=sqrt((X3(1)*a*l(3))/(2*pi*eta(3)));

for t=2:(n1+1)

r12=r12poc*(t==2)+(r12poc-0.01)*(t==3)+(r12poc-0.01-0.01)*(t==4)+(r12poc-0.09-0.09-0.06)*(t==5)+(r12poc-0.09-0.09-0.08-0.08)*(t==6)+(r12poc-0.04-0.06-0.08-0.12-0.08)*(t==7)+(r12poc-0.04-0.06-0.08-0.12-0.08-0.06)*(t==8)+(r12poc-0.04-0.06-0.08-0.12-0.08-0.06-(t-8)*0.02)*(t>8);

r11=1-r12;

rvar(t-1)=r12;

l(1)=alfa(1)*(t-2)+beta(1);

l(2)=alfa(2)*(t-2)+beta(2);

l(3)=alfa(3)*(t-2)+beta(3);

l(4)=alfa(4)*(t-2)+beta(4);

eta(1)=et(1)+(t-1)*ni_11;

eta(2)=et(2)+(t-1)*ni_12;

eta(3)=et(3)+(t-1)*ni_13;

eta(4)=et(4)+(t-1)*ni_14;

apom(1)=a*l(1);

apom(2)=a*l(2);

apom(3)=a*l(3);

apom(4)=a*l(4);

rpom=X(t-1)/(eta(1)/apom(1)+eta(2)/apom(2)+eta(3)/apom(3)+eta(4)/apom(4));

X1(t-1)=floor((eta(1)/apom(1))*rpom);

X2(t-1)=floor((eta(2)/apom(2))*rpom);

X3(t-1)=floor((eta(3)/apom(3))*rpom);

X4(t-1)=floor((eta(4)/apom(4))*rpom);

l(1)=l(1)+alfa(1);

l(2)=l(2)+alfa(2);

l(3)=l(3)+alfa(3);

l(4)=l(4)+alfa(4);

apom(1)=a*l(1);

apom(2)=a*l(2);

apom(3)=a*l(3);

apom(4)=a*l(4);

etapo1(t-1)=eta(1);

etapo2(t-1)=eta(2);

etapo3(t-1)=eta(3);

etapo4(t-1)=eta(4);

apo1(t-1)=a*l(1);

apo2(t-1)=a*l(2);

apo3(t-1)=a*l(3);

apo4(t-1)=a*l(4);

avar1(t-1)=apom(1);

avar2(t-1)=apom(2);

avar4(t-1)=apom(4);

avar3(t-1)=apom(3);

for i=1:4

c(i)=2*eta(i)*ro*w/(a*l(i));

d(i)=alfa(i)/(l(i));

end

O(1)=0;

O(2)=0;

O(3)=0;

for i=1:X1(t-1)

pom1=rand();

if (pom1>p10) && (pom1<=p10+p11)

O(1)=O(1)+1;

end

if (pom1>p10+p11)

O(1)=O(1)+2;

end

end

for i=1:X2(t-1)

pom1=rand();

if (pom1>q10) && (pom1<=q10+q11)

O(2)=O(2)+1;

end

if (pom1>q10+q11)

O(2)=O(2)+2;

end

end

for i=1:X3(t-1)

pom1=rand();

if (pom1>r10) && (pom1<=r10+r11)

O(3)=O(3)+1;

end

if (pom1>r10+r11)

O(3)=O(3)+2;

end

end

rpom=X(t-1)/(eta_1/apom(1)+eta_2/apom(2)+eta_3/apom(3)+eta_4/apom(4));

v=rpom*delta1*(2-delta1)/(2*ro*w);

u=1/(1+c(1)+c(2)+c(3)+c(4))*(d(1)*X1(t-1)+d(2)*X2(t-1)+d(3)*X3(t-1)+d(4)*X4(t-1)+O(1)-X1(t-1)+O(2)-X2(t-1)+O(3)-X3(t-1)-v);

xpom=0;

ypom=0;

zpom=0;

un=floor(u);

xn=floor(xpom);

yn=floor(ypom);

zn=floor(zpom);

X1(t)=O(1)+xn;

X2(t)=O(2)-xn-yn;

X3(t)=O(3)-floor(u);

X4(t)=0;

R(t)=sqrt((X3(t)*a*l(3))/(2*pi*eta(3)));

X(t)=X1(t)+X2(t)+X3(t)+X4(t);

X21(t-1)=xn;

X23(t-1)=yn;

X34(t-1)=zn;

XEbes(t-1)=floor(u+v);

X1pom(t)=X1(t);

X2pom(t)=X2(t);

X3pom(t)=X3(t);

X4pom(t)=X4(t);

end

n=n1;

var(1,:)=X;

var(2,:)=R;

var(3,1:(n+1))=X1pom(1:(n+1));

var(4,1:(n+1))=X2pom(1:(n+1));

var(5,1:(n+1))=X3pom(1:(n+1));

var(6,1:(n+1))=X4pom(1:(n+1));

var(7,1:n)=X21;

var(8,1:n)=X23;

var(9,1:n)=X34;

var(10,1:n)=XEbes;

var(11,1:n)=rvar;

var(12,1:n)=avar1(1:n);

var(13,1:n)=avar2(1:n);

var(14,1:n)=avar3(1:n);

var(15,1:n)=avar4(1:n);

var(16,1:n)=etapo1(1:n);

var(17,1:n)=etapo2(1:n);

var(18,1:n)=etapo3(1:n);

var(19,1:n)=etapo4(1:n);

end

Subprogram mark7_p_sapoc_2.m:

function var=mark7_p_sapoc_2(N1,N2,N3,N4,beta_1,beta_2,beta_3,beta_4)

alfa_1=0;

alfa_2=13/600;

alfa_3=1/75;

alfa_4=1/200;

draw1=mark7p1_p3_stlouis(3,7.8,1.9,200,0,0,1,0,0,0.14,-0.15,0.01,beta_1,beta_2,beta_3,beta_4,alfa_1,alfa_2,alfa_3,alfa_4,N1,N2,N3,N4,0,0,0,0,0.83,0.17,0,0.7,0.3,0);

var(1,1:4)=draw1(1,:);

var(2,1:4)=draw1(2,:);

var(3,1:4)=draw1(3,:);

var(4,1:4)=draw1(4,:);

var(5,1:4)=draw1(5,:);

var(6,1:4)=draw1(6,:);

var(7,1:3)=draw1(7,1:3);

var(8,1:3)=draw1(8,1:3);

var(9,1:3)=draw1(9,1:3);

var(10,1:3)=draw1(10,1:3);

var(11,1:3)=draw1(11,1:3);

var(12,1:3)=0;

var(13,1:3)=0;

var(14,1:3)=draw1(14,1:3);

var(15,1:3)=0;

var(16,1:3)=draw1(16,1:3);

var(17,1:3)=draw1(17,1:3);

var(18,1:3)=draw1(18,1:3);

var(19,1:3)=draw1(19,1:3);

beta_1=3*alfa_1+beta_1;

beta_2=3*alfa_2+beta_2;

beta_3=3*alfa_3+beta_3;

beta_4=3*alfa_4+beta_4;

alfa_1=0;

alfa_2=13/600;

alfa_3=1/75;

alfa_4=1/200;

draw2=mark7p1_p3_stlouis2(3,9,2,200,0,0.42,0.55,0.03,0,0.03,-0.05,0.02,beta_1,beta_2,beta_3,beta_4,alfa_1,alfa_2,alfa_3,alfa_4,draw1(3,4),draw1(4,4),draw1(5,4),draw1(6,4),0,0,0,0,0.84,0.16,0,0.7,0.3,0);

var(1,5:7)=draw2(1,2:4);

var(2,5:7)=draw2(2,2:4);

var(3,5:7)=draw2(3,2:4);

var(4,5:7)=draw2(4,2:4);

var(5,5:7)=draw2(5,2:4);

var(6,5:7)=draw2(6,2:4);

var(7,4:6)=draw2(7,1:3);

var(8,4:6)=draw2(8,1:3);

var(9,4:6)=draw2(9,1:3);

var(10,4:6)=draw2(10,1:3);

var(11,4:6)=draw2(11,1:3);

var(12,4:6)=0;

var(13,4:6)=draw2(13,1:3);

var(14,4:6)=draw2(14,1:3);

var(15,4:6)=draw2(15,1:3);

var(16,4:6)=draw2(16,1:3);

var(17,4:6)=draw2(17,1:3);

var(18,4:6)=draw2(18,1:3);

var(19,4:6)=draw2(19,1:3);

beta_1=3*alfa_1+beta_1;

beta_2=3*alfa_2+beta_2;

beta_3=3*alfa_3+beta_3;

beta_4=3*alfa_4+beta_4;

alfa_1=0;

alfa_2=1/60;

alfa_3=1/60;

alfa_4=1/240;

draw3= mark7p1_p3_stlouis2(1,9,2,200,0,0.51,0.40,0.09,0,0.01,-0.01,0,beta_1,beta_2,beta_3,beta_4,alfa_1,alfa_2,alfa_3,alfa_4,draw2(3,4),draw2(4,4),draw2(5,4),draw2(6,4),0,0,0,0,0.87,0.13,0,0.73,0.27,0);

var(1,8)=draw3(1,2);

var(2,8)=draw3(2,2);

var(3,8)=draw3(3,2);

var(4,8)=draw3(4,2);

var(5,8)=draw3(5,2);

var(6,8)=draw3(6,2);

var(7,7)=draw3(7,1);

var(8,7)=draw3(8,1);

var(9,7)=draw3(9,1);

var(10,7)=draw3(10,1);

var(11,7)=draw3(11,1);

var(12,7)=0;

var(13,7)=draw3(13,1);

var(14,7)=draw3(14,1);

var(15,7)=draw3(15,1);

var(16,7)=draw3(16,1);

var(17,7)=draw3(17,1);

var(18,7)=draw3(18,1);

var(19,7)=draw3(19,1);

beta_1=alfa_1+beta_1;

beta_2=alfa_2+beta_2;

beta_3=alfa_3+beta_3;

beta_4=alfa_4+beta_4;

alfa_1=0;

alfa_2=1/60;

alfa_3=1/60;

alfa_4=1/240;

draw4=mark7p1_p3_stlouis2(5,10,2,200,0,0.52,0.39,0.09,0,0.01,-0.01,0,beta_1,beta_2,beta_3,beta_4,alfa_1,alfa_2,alfa_3,alfa_4,draw3(3,2),draw3(4,2),draw3(5,2),draw3(6,2),0,0,0,0,0.93,0.07,0,0.8,0.2,0);

var(1,9:13)=draw4(1,2:6);

var(2,9:13)=draw4(2,2:6);

var(3,9:13)=draw4(3,2:6);

var(4,9:13)=draw4(4,2:6);

var(5,9:13)=draw4(5,2:6);

var(6,9:13)=draw4(6,2:6);

var(7,8:12)=draw4(7,1:5);

var(8,8:12)=draw4(8,1:5);

var(9,8:12)=draw4(9,1:5);

var(10,8:12)=draw4(10,1:5);

var(11,8:12)=draw4(11,1:5);

var(12,8:12)=0;

var(13,8:12)=draw4(13,1:5);

var(14,8:12)=draw4(14,1:5);

var(15,8:12)=draw4(15,1:5);

var(16,8:12)=draw4(16,1:5);

var(17,8:12)=draw4(17,1:5);

var(18,8:12)=draw4(18,1:5);

var(19,8:12)=draw4(19,1:5);

beta_1=9/20;

beta_2=5*alfa_2+beta_2;

beta_3=5*alfa_3+beta_3;

beta_4=5*alfa_4+beta_4;

alfa_1=1/40;

alfa_2=3/200;

alfa_3=1/200;

alfa_4=1/400;

draw5=mark7p1_p3_stlouis2(4,10,2,200,0,0.57,0.34,0.09,0.05,-0.04,-0.01,0,beta_1,beta_2,beta_3,beta_4,alfa_1,alfa_2,alfa_3,alfa_4,draw4(3,6),draw4(4,6),draw4(5,6),draw4(6,6),0,1,0,0,0.93,0.07,0,0.81,0.19,0);

var(1,14:17)=draw5(1,2:5);

var(2,14:17)=draw5(2,2:5);

var(3,14:17)=draw5(3,2:5);

var(4,14:17)=draw5(4,2:5);

var(5,14:17)=draw5(5,2:5);

var(6,14:17)=draw5(6,2:5);

var(7,13:16)=draw5(7,1:4);

var(8,13:16)=draw5(8,1:4);

var(9,13:16)=draw5(9,1:4);

var(10,13:16)=draw5(10,1:4);

var(11,13:16)=draw5(11,1:4);

var(12,13:16)=0;

var(13,13:16)=draw5(13,1:4);

var(14,13:16)=draw5(14,1:4);

var(15,13:16)=draw5(15,1:4);

var(16,13:16)=draw5(16,1:4);

var(17,13:16)=draw5(17,1:4);

var(18,13:16)=draw5(18,1:4);

var(19,13:16)=draw5(19,1:4);

beta_1=4*alfa_1+beta_1;

beta_2=4*alfa_2+beta_2;

beta_3=4*alfa_3+beta_3;

beta_4=4*alfa_4+beta_4;

alfa_1=17/400;

alfa_2=1/100;

alfa_3=1/100;

alfa_4=1/400;

draw6=mark7p1_p3_stlouis2(4,10,2,200,0.20,0.41,0.3,0.09,0.03,-0.02,-0.01,0,beta_1,beta_2,beta_3,beta_4,alfa_1,alfa_2,alfa_3,alfa_4,draw5(3,5),draw5(4,5),draw5(5,5),draw5(6,5),0,1,0,0,0.94,0.06,0,0.85,0.15,0);

var(1,18:21)=draw6(1,2:5);

var(2,18:21)=draw6(2,2:5);

var(3,18:21)=draw6(3,2:5);

var(4,18:21)=draw6(4,2:5);

var(5,18:21)=draw6(5,2:5);

var(6,18:21)=draw6(6,2:5);

var(7,17:20)=draw6(7,1:4);

var(8,17:20)=draw6(8,1:4);

var(9,17:20)=draw6(9,1:4);

var(10,17:20)=draw6(10,1:4);

var(11,17:20)=draw6(11,1:4);

var(12,17:20)=draw6(12,1:4);

var(13,17:20)=draw6(13,1:4);

var(14,17:20)=draw6(14,1:4);

var(15,17:20)=draw6(15,1:4);

var(16,17:20)=draw6(16,1:4);

var(17,17:20)=draw6(17,1:4);

var(18,17:20)=draw6(18,1:4);

var(19,17:20)=draw6(19,1:4);

beta_1=4*alfa_1+beta_1;

beta_2=4*alfa_2+beta_2;

beta_3=4*alfa_3+beta_3;

beta_4=4*alfa_4+beta_4;

alfa_1=1/100;

alfa_2=1/80;

alfa_3=9/800;

alfa_4=3/800;

draw7= mark7p1_p3_2(8,10,2,200,0.32,0.33,0.26,0.09,0,0,0,0,beta_1,beta_2,beta_3,beta_4,alfa_1,alfa_2,alfa_3,alfa_4,draw6(3,5),draw6(4,5),draw6(5,5),draw6(6,5),0,1,0,0,0.94,0.06,0,0.86,0.14,0);

var(1,22:29)=draw7(1,2:9);

var(2,22:29)=draw7(2,2:9);

var(3,22:29)=draw7(3,2:9);

var(4,22:29)=draw7(4,2:9);

var(5,22:29)=draw7(5,2:9);

var(6,22:29)=draw7(6,2:9);

var(7,21:28)=draw7(7,1:8);

var(8,21:28)=draw7(8,1:8);

var(9,21:28)=draw7(9,1:8);

var(10,21:28)=draw7(10,1:8);

var(11,21:28)=draw7(11,1:8);

var(12,21:28)=draw7(12,1:8);

var(13,21:28)=draw7(13,1:8);

var(14,21:28)=draw7(14,1:8);

var(15,21:28)=draw7(15,1:8);

var(16,21:28)=draw7(16,1:8);

var(17,21:28)=draw7(17,1:8);

var(18,21:28)=draw7(18,1:8);

var(19,21:28)=draw7(19,1:8);

var(20,1)=draw7(3,9);

var(20,2)=draw7(4,9);

var(20,3)=draw7(5,9);

var(20,4)=draw7(6,9);

var(20,5)=8*alfa_1+beta_1;

var(20,6)=8*alfa_2+beta_2;

var(20,7)=8*alfa_3+beta_3;

var(20,8)=8*alfa_4+beta_4;

end

Subprogram mark7p1_p3_stlouis.m:

function var=mark7p1_p3_stlouis(n1,ro,w,a,eta_1,eta_2,eta_3,eta_4,ni_11,ni_12,ni_13,ni_14,beta_1,beta_2,beta_3,beta_4,alfa_11,alfa_12,alfa_13,alfa_14,N1,N2,N3,N4,p10,p11,p12,q10,q11,q12,r10,r11,r12,delta1)

X1(1)=N1;

X2(1)=N2;

X3(1)=N3;

X4(1)=N4;

X(1)=X1(1)+X2(1)+X3(1)+X4(1);

beta(1)=beta_1;

beta(2)=beta_2;

beta(3)=beta_3;

beta(4)=beta_4;

alfa(1)=alfa_11;

alfa(2)=alfa_12;

alfa(3)=alfa_13;

alfa(4)=alfa_14;

l(3)=beta(3);

A(1)=N3*a*beta_3;

R(1)=sqrt(A(1)/(2*pi));

for t=2:(n1+1)

rvar(t-1)=r12;

l(1)=alfa(1)*(t-2)+beta(1);

l(2)=alfa(2)*(t-2)+beta(2);

l(3)=alfa(3)*(t-2)+beta(3);

l(4)=alfa(4)*(t-2)+beta(4);

eta(1)=eta_1+(t-2)*ni_11;

eta(2)=eta_2+(t-2)*ni_12;

eta(3)=eta_3+(t-2)*ni_13;

eta(4)=eta_4+(t-2)*ni_14;

apom(1)=a*l(1);

apom(2)=a*l(2);

apom(3)=a*l(3);

apom(4)=a*l(4);

etaukupno=eta(1)/apom(1)+eta(2)/apom(2)+eta(3)/apom(3)+eta(4)/apom(4);

O(1)=0;

O(2)=0;

O(3)=0;

for i=1:X1(t-1)

pom1=rand();

if (pom1>p10) && (pom1<=p10+p11)

O(1)=O(1)+1;

end

if (pom1>p10+p11)

O(1)=O(1)+2;

end

end

for i=1:X2(t-1)

pom1=rand();

if (pom1>q10) && (pom1<=q10+q11)

O(2)=O(2)+1;

end

if (pom1>q10+q11)

O(2)=O(2)+2;

end

end

for i=1:X3(t-1)

pom1=rand();

if (pom1>r10) && (pom1<=r10+r11)

O(3)=O(3)+1;

end

if (pom1>r10+r11)

O(3)=O(3)+2;

end

end

l(1)=l(1)+alfa(1);

l(2)=l(2)+alfa(2);

l(3)=l(3)+alfa(3);

l(4)=l(4)+alfa(4);

apom(1)=a*l(1);

apom(2)=a*l(2);

apom(3)=a*l(3);

apom(4)=a*l(4);

avar1(t-1)=apom(1);

avar2(t-1)=apom(2);

avar3(t-1)=apom(3);

avar4(t-1)=apom(4);

eta(1)=eta(1)+ni_11;

eta(2)=eta(2)+ni_12;

eta(3)=eta(3)+ni_13;

eta(4)=eta(4)+ni_14;

etavar1(t-1)=eta(1);

etavar2(t-1)=eta(2);

etavar3(t-1)=eta(3);

etavar4(t-1)=eta(4);

etaukupnonovi=eta(1)/apom(1)+eta(2)/apom(2)+eta(3)/apom(3)+eta(4)/apom(4);

u=(1/(1+2*ro*w*etaukupnonovi))*(O(1)+O(2)+O(3)+X4(t-1)-(etaukupnonovi/etaukupno)*X(t-1));

A(t)=A(t-1)+2*u*ro*w;

X1(t)=floor(eta(1)*A(t)/apom(1));

X2(t)=floor(eta(2)*A(t)/apom(2));

X3(t)=floor(eta(3)*A(t)/apom(3));

X4(t)=floor(eta(4)*A(t)/apom(4));

X(t)=X1(t)+X2(t)+X3(t)+X4(t);

R(t)=sqrt(A(t)/(2*pi));

XEbes(t-1)=floor(u);

end

n=n1;

var(1,:)=X;

var(2,:)=R;

var(3,1:(n+1))=X1(1:(n+1));

var(4,1:(n+1))=X2(1:(n+1));

var(5,1:(n+1))=X3(1:(n+1));

var(6,1:(n+1))=X4(1:(n+1));

var(7,1:n)=0;

var(8,1:n)=0;

var(9,1:n)=0;

var(10,1:n)=XEbes;

var(11,1:n)=rvar;

var(12,1:n)=avar1(1:n);

var(13,1:n)=avar2(1:n);

var(14,1:n)=avar3(1:n);

var(15,1:n)=avar4(1:n);

var(16,1:n)=etavar1;

var(17,1:n)=etavar2;

var(18,1:n)=etavar3;

var(19,1:n)=etavar4;

end

Subprogram mark7p1_p3_stlouis2.m:

function var=mark7p1_p3_stlouis2(n1,ro,w,a,eta_1,eta_2,eta_3,eta_4,ni_11,ni_12,ni_13,ni_14,beta_1,beta_2,beta_3,beta_4,alfa_11,alfa_12,alfa_13,alfa_14,N1,N2,N3,N4,p10,p11,p12,q10,q11,q12,r10,r11,r12,delta1)

X1(1)=N1;

X2(1)=N2;

X3(1)=N3;

X4(1)=N4;

X(1)=X1(1)+X2(1)+X3(1)+X4(1);

beta(1)=beta_1;

beta(2)=beta_2;

beta(3)=beta_3;

beta(4)=beta_4;

alfa(1)=alfa_11;

alfa(2)=alfa_12;

alfa(3)=alfa_13;

alfa(4)=alfa_14;

l(3)=beta(3);

A(1)=N1*a*beta_1+N2*a*beta_2+N3*a*beta_3+N4*a*beta_4;

R(1)=sqrt(A(1)/(2*pi));

for t=2:(n1+1)

rvar(t-1)=r12;

l(1)=alfa(1)*(t-2)+beta(1);

l(2)=alfa(2)*(t-2)+beta(2);

l(3)=alfa(3)*(t-2)+beta(3);

l(4)=alfa(4)*(t-2)+beta(4);

eta(1)=eta_1+(t-2)*ni_11;

eta(2)=eta_2+(t-2)*ni_12;

eta(3)=eta_3+(t-2)*ni_13;

eta(4)=eta_4+(t-2)*ni_14;

apom(1)=a*l(1);

apom(2)=a*l(2);

apom(3)=a*l(3);

apom(4)=a*l(4);

etaukupno=eta(1)/apom(1)+eta(2)/apom(2)+eta(3)/apom(3)+eta(4)/apom(4);

O(1)=0;

O(2)=0;

O(3)=0;

for i=1:X1(t-1)

pom1=rand();

if (pom1>p10) && (pom1<=p10+p11)

O(1)=O(1)+1;

end

if (pom1>p10+p11)

O(1)=O(1)+2;

end

end

for i=1:X2(t-1)

pom1=rand();

if (pom1>q10) && (pom1<=q10+q11)

O(2)=O(2)+1;

end

if (pom1>q10+q11)

O(2)=O(2)+2;

end

end

for i=1:X3(t-1)

pom1=rand();

if (pom1>r10) && (pom1<=r10+r11)

O(3)=O(3)+1;

end

if (pom1>r10+r11)

O(3)=O(3)+2;

end

end

l(1)=l(1)+alfa(1);

l(2)=l(2)+alfa(2);

l(3)=l(3)+alfa(3);

l(4)=l(4)+alfa(4);

apom(1)=a*l(1);

apom(2)=a*l(2);

apom(3)=a*l(3);

apom(4)=a*l(4);

avar1(t-1)=apom(1);

avar2(t-1)=apom(2);

avar3(t-1)=apom(3);

avar4(t-1)=apom(4);

eta(1)=eta(1)+ni_11;

eta(2)=eta(2)+ni_12;

eta(3)=eta(3)+ni_13;

eta(4)=eta(4)+ni_14;

etavar1(t-1)=eta(1);

etavar2(t-1)=eta(2);

etavar3(t-1)=eta(3);

etavar4(t-1)=eta(4);

etaukupnonovi=eta(1)/apom(1)+eta(2)/apom(2)+eta(3)/apom(3)+eta(4)/apom(4);

u=(1/(1+2*ro*w*etaukupnonovi))*(O(1)+O(2)+O(3)+X4(t-1)-(etaukupnonovi/etaukupno)*X(t-1));

A(t)=A(t-1)+2*u*ro*w;

X1(t)=floor(eta(1)*A(t)/apom(1));

X2(t)=floor(eta(2)*A(t)/apom(2));

X3(t)=floor(eta(3)*A(t)/apom(3));

X4(t)=floor(eta(4)*A(t)/apom(4));

X(t)=X1(t)+X2(t)+X3(t)+X4(t);

R(t)=sqrt(A(t)/(2*pi));

XEbes(t-1)=floor(u);

end

n=n1;

var(1,:)=X;

var(2,:)=R;

var(3,1:(n+1))=X1(1:(n+1));

var(4,1:(n+1))=X2(1:(n+1));

var(5,1:(n+1))=X3(1:(n+1));

var(6,1:(n+1))=X4(1:(n+1));

var(7,1:n)=0;

var(8,1:n)=0;

var(9,1:n)=0;

var(10,1:n)=XEbes;

var(11,1:n)=rvar;

var(12,1:n)=avar1(1:n);

var(13,1:n)=avar2(1:n);

var(14,1:n)=avar3(1:n);

var(15,1:n)=avar4(1:n);

var(16,1:n)=etavar1;

var(17,1:n)=etavar2;

var(18,1:n)=etavar3;

var(19,1:n)=etavar4;

end

Subprogram mark7p1_p3_2.m:

function var=mark7p1_p3_2(n1,ro,w,a,eta_1,eta_2,eta_3,eta_4,ni_11,ni_12,ni_13,ni_14,beta_1,beta_2,beta_3,beta_4,alfa_11,alfa_12,alfa_13,alfa_14,N1,N2,N3,N4,p10,p11,p12,q10,q11,q12poc,r10,r11,r12poc,delta1)

X1(1)=N1;

X2(1)=N2;

X3(1)=N3;

X4(1)=N4;

X1pom(1)=X1(1);

X2pom(1)=X2(1);

X3pom(1)=X3(1);

X4pom(1)=X4(1);

X(1)=X1(1)+X2(1)+X3(1)+X4(1);

et(1)=eta_1;

et(2)=eta_2;

et(3)=eta_3;

et(4)=eta_4;

beta(1)=beta_1;

beta(2)=beta_2;

beta(3)=beta_3;

beta(4)=beta_4;

alfa(1)=alfa_11;

alfa(2)=alfa_12;

alfa(3)=alfa_13;

alfa(4)=alfa_14;

l(3)=beta(3);

eta(3)=et(3);

R(1)=sqrt((X3(1)*a*l(3))/(2*pi*eta(3)));

for t=2:(n1+1)

r12=r12poc*(t==2)+0.14*(t==3)+0.12*(t==4)+0.12*(t==5)+0.1*(t==6)+0.1*(t==7)+0.09*(t==8)+0.09*(t==9);

r11=1-r12;

q12=q12poc*(t==2)+0.06*(t==3)+0.06*(t==4)+0.05*(t==5)+0.05*(t==6)+0.05*(t==7)+0.05*(t==8)+0.05*(t==9);

q11=1-q12;

l(1)=alfa(1)*(t-2)+beta(1);

l(2)=alfa(2)*(t-2)+beta(2);

l(3)=alfa(3)*(t-2)+beta(3);

l(4)=alfa(4)*(t-2)+beta(4);

eta(1)=et(1)+(t-1)*ni_11;

eta(2)=et(2)+(t-1)*ni_12;

eta(3)=et(3)+(t-1)*ni_13;

eta(4)=et(4)+(t-1)*ni_14;

apom(1)=a*l(1);

apom(2)=a*l(2);

apom(3)=a*l(3);

apom(4)=a*l(4);

etavar1(t-1)=eta(1);

etavar2(t-1)=eta(2);

etavar3(t-1)=eta(3);

etavar4(t-1)=eta(4);

rpom=X(t-1)/(eta(1)/apom(1)+eta(2)/apom(2)+eta(3)/apom(3)+eta(4)/apom(4));

X1(t-1)=floor((eta(1)/apom(1))*rpom);

X2(t-1)=floor((eta(2)/apom(2))*rpom);

X3(t-1)=floor((eta(3)/apom(3))*rpom);

X4(t-1)=floor((eta(4)/apom(4))*rpom);

l(1)=l(1)+alfa(1);

l(2)=l(2)+alfa(2);

l(3)=l(3)+alfa(3);

l(4)=l(4)+alfa(4);

apom(1)=a*l(1);

apom(2)=a*l(2);

apom(3)=a*l(3);

apom(4)=a*l(4);

avar1(t-1)=apom(1);

avar2(t-1)=apom(2);

avar3(t-1)=apom(3);

avar4(t-1)=apom(4);

etapo1(t-1)=eta(1);

etapo2(t-1)=eta(2);

etapo3(t-1)=eta(3);

etapo4(t-1)=eta(4);

apo1(t-1)=a*l(1);

apo2(t-1)=a*l(2);

apo3(t-1)=a*l(3);

apo4(t-1)=a*l(4);

for i=1:4

c(i)=2*eta(i)*ro*w/(a*l(i));

d(i)=alfa(i)/(l(i));

end

O(1)=0;

O(2)=0;

O(3)=0;

for i=1:X1(t-1)

pom1=rand();

if (pom1>p10) && (pom1<=p10+p11)

O(1)=O(1)+1;

end

if (pom1>p10+p11)

O(1)=O(1)+2;

end

end

for i=1:X2(t-1)

pom1=rand();

if (pom1>q10) && (pom1<=q10+q11)

O(2)=O(2)+1;

end

if (pom1>q10+q11)

O(2)=O(2)+2;

end

end

for i=1:X3(t-1)

pom1=rand();

if (pom1>r10) && (pom1<=r10+r11)

O(3)=O(3)+1;

end

if (pom1>r10+r11)

O(3)=O(3)+2;

end

end

rpom=X(t-1)/(eta_1/apom(1)+eta_2/apom(2)+eta_3/apom(3)+eta_4/apom(4));

v=rpom*delta1*(2-delta1)/(2*ro*w);

u=1/(1+c(1)+c(2)+c(3)+c(4))*(d(1)*X1(t-1)+d(2)*X2(t-1)+d(3)*X3(t-1)+d(4)*X4(t-1)+O(1)-X1(t-1)+O(2)-X2(t-1)+O(3)-X3(t-1)-v);

xpom=c(1)*u-(O(1)-X1(t-1))-d(1)*X1(t-1);

ypom=u*(1+c(3)+c(4))-d(3)*X3(t-1)-d(4)*X4(t-1)-(O(3)-X3(t-1))+v;

zpom=u*(1+c(4))-d(4)*X4(t-1)+v;

un=floor(u);

xn=floor(xpom);

yn=floor(ypom);

zn=floor(zpom);

X1(t)=O(1)+xn;

X2(t)=O(2)-xn-yn;

X3(t)=O(3)-zn+yn;

X4(t)=X4(t-1)+zn-floor(u+v);

R(t)=sqrt((X3(t)*a*l(3))/(2*pi*eta(3)));

X(t)=X1(t)+X2(t)+X3(t)+X4(t);

X21(t-1)=xn;

X23(t-1)=yn;

X34(t-1)=zn;

XEbes(t-1)=floor(u+v);

X1pom(t)=X1(t);

X2pom(t)=X2(t);

X3pom(t)=X3(t);

X4pom(t)=X4(t);

end

n=n1;

var(1,:)=X;

var(2,:)=R;

var(3,1:(n+1))=X1pom(1:(n+1));

var(4,1:(n+1))=X2pom(1:(n+1));

var(5,1:(n+1))=X3pom(1:(n+1));

var(6,1:(n+1))=X4pom(1:(n+1));

var(7,1:n)=X21;

var(8,1:n)=X23;

var(9,1:n)=X34;

var(10,1:n)=XEbes;

var(11,1:n)=r12;

var(12,1:n)=avar1;

var(13,1:n)=avar2;

var(14,1:n)=avar3;

var(15,1:n)=avar4;

var(16,1:n)=etavar1;

var(17,1:n)=etavar2;

var(18,1:n)=etavar3;

var(19,1:n)=etavar4;

end

Subprogram allfhases_2.m:

function var=allfhases_2(n1,n2,n3,n4,n5,n6,n7,n8,ro,w,a,eta_1,eta_2,eta_3,eta_4,eta_5,eta_6,eta_7,eta_8,ni_11,ni_12,ni_13,ni_14,ni_21,ni_22,ni_23,ni_24,ni_31,ni_32,ni_33,ni_34,ni_41,ni_42,ni_43,ni_44,ni_51,ni_52,ni_53,ni_54,ni_61,ni_62,ni_63,ni_64,ni_71,ni_72,ni_73,ni_74,ni_81,ni_82,ni_83,ni_84,beta_1,beta_2,beta_3,beta_4,alfa_11,alfa_12,alfa_13,alfa_14,alfa_21,alfa_22,alfa_23,alfa_24,alfa_31,alfa_32,alfa_33,alfa_34,alfa_41,alfa_42,alfa_43,alfa_44,alfa_51,alfa_52,alfa_53,alfa_54,alfa_61,alfa_62,alfa_63,alfa_64,alfa_71,alfa_72,alfa_73,alfa_74,alfa_81,alfa_82,alfa_83,alfa_84,N1,N2,N3,N4,p10,p11,p12,p20,p21,p22,p30,p31,p32,p40,p41,p42,p50,p51,p52,p60,p61,p62,p70,p71,p72,p80,p81,p82,q10,q11,q12,q20,q21,q22,q30,q31,q32,q40,q41,q42,q50,q51,q52,q60,q61,q62,q70,q71,q72,q80,q81,q82,r10,r11,r12,r20,r21,r22,r30,r31,r32,r40,r41,r42,r50,r51,r52,r60,r61,r62,r70,r71,r72,r80,r81,r82,delta1,delta2,delta3,delta4,delta5,delta6,delta7,delta8)

X1(1)=N1;

X2(1)=N2;

X3(1)=N3;

X4(1)=N4;

X1pom(1)=X1(1);

X2pom(1)=X2(1);

X3pom(1)=X3(1);

X4pom(1)=X4(1);

X(1)=X1(1)+X2(1)+X3(1)+X4(1);

et(1)=eta_1;

et(2)=eta_2;

et(3)=eta_3;

et(4)=eta_4;

beta(1)=beta_1;

beta(2)=beta_2;

beta(3)=beta_3;

beta(4)=beta_4;

alfa(1)=alfa_11;

alfa(2)=alfa_12;

alfa(3)=alfa_13;

alfa(4)=alfa_14;

for t=2:(n1+1)

l(1)=alfa(1)*(t-2)+beta(1);

l(2)=alfa(2)*(t-2)+beta(2);

l(3)=alfa(3)*(t-2)+beta(3);

l(4)=alfa(4)*(t-2)+beta(4);

eta(1)=et(1)+(t-1)*ni_11;

eta(2)=et(2)+(t-1)*ni_12;

eta(3)=et(3)+(t-1)*ni_13;

eta(4)=et(4)+(t-1)*ni_14;

apom(1)=a*l(1);

apom(2)=a*l(2);

apom(3)=a*l(3);

apom(4)=a*l(4);

rpom=X(t-1)/(eta(1)/apom(1)+eta(2)/apom(2)+eta(3)/apom(3)+eta(4)/apom(4));

X1(t-1)=floor((eta(1)/apom(1))*rpom);

X2(t-1)=floor((eta(2)/apom(2))*rpom);

X3(t-1)=floor((eta(3)/apom(3))*rpom);

X4(t-1)=floor((eta(4)/apom(4))*rpom);

l(1)=l(1)+alfa(1);

l(2)=l(2)+alfa(2);

l(3)=l(3)+alfa(3);

l(4)=l(4)+alfa(4);

apom(1)=a*l(1);

apom(2)=a*l(2);

apom(3)=a*l(3);

apom(4)=a*l(4);

etapo1(t-1)=eta(1);

etapo2(t-1)=eta(2);

etapo3(t-1)=eta(3);

etapo4(t-1)=eta(4);

apo1(t-1)=a*l(1);

apo2(t-1)=a*l(2);

apo3(t-1)=a*l(3);

apo4(t-1)=a*l(4);

for i=1:4

c(i)=2*eta(i)*ro*w/(a*l(i));

d(i)=alfa(i)/(l(i));

end

O(1)=0;

O(2)=0;

O(3)=0;

for i=1:X1(t-1)

pom1=rand();

if (pom1>p10) && (pom1<=p10+p11)

O(1)=O(1)+1;

end

if (pom1>p10+p11)

O(1)=O(1)+2;

end

end

for i=1:X2(t-1)

pom1=rand();

if (pom1>q10) && (pom1<=q10+q11)

O(2)=O(2)+1;

end

if (pom1>q10+q11)

O(2)=O(2)+2;

end

end

for i=1:X3(t-1)

pom1=rand();

if (pom1>r10) && (pom1<=r10+r11)

O(3)=O(3)+1;

end

if (pom1>r10+r11)

O(3)=O(3)+2;

end

end

rpom=X(t-1)/(eta_1/apom(1)+eta_2/apom(2)+eta_3/apom(3)+eta_4/apom(4));

v=rpom*delta1*(2-delta1)/(2*ro*w);

u=1/(1+c(1)+c(2)+c(3)+c(4))*(d(1)*X1(t-1)+d(2)*X2(t-1)+d(3)*X3(t-1)+d(4)*X4(t-1)+O(1)-X1(t-1)+O(2)-X2(t-1)+O(3)-X3(t-1)-v);

xpom=c(1)*u-(O(1)-X1(t-1))-d(1)*X1(t-1);

ypom=u*(1+c(3)+c(4))-d(3)*X3(t-1)-d(4)*X4(t-1)-(O(3)-X3(t-1))+v;

zpom=u*(1+c(4))-d(4)*X4(t-1)+v;

un=floor(u);

xn=floor(xpom);

yn=floor(ypom);

zn=floor(zpom);

X1(t)=O(1)+xn;

X2(t)=O(2)-xn-yn;

X3(t)=O(3)-zn+yn;

X4(t)=X4(t-1)+zn-floor(u+v);

R(t)=sqrt((X1(t)*a*l(1))/(2*pi*eta(1)));

X(t)=X1(t)+X2(t)+X3(t)+X4(t);

X21(t-1)=xn;

X23(t-1)=yn;

X34(t-1)=zn;

XEbes(t-1)=floor(u+v);

X1pom(t)=X1(t);

X2pom(t)=X2(t);

X3pom(t)=X3(t);

X4pom(t)=X4(t);

end

beta(1)=n1*alfa_11+beta_1;

beta(2)=n1*alfa_12+beta_2;

beta(3)=n1*alfa_13+beta_3;

beta(4)=n1*alfa_14+beta_4;

alfa(1)=alfa_21;

alfa(2)=alfa_22;

alfa(3)=alfa_23;

alfa(4)=alfa_24;

et(1)=n1*ni_11+et(1);

et(2)=n1*ni_12+et(2);

et(3)=n1*ni_13+et(3);

et(4)=n1*ni_14+et(4);

for t=(2+n1):(n1+n2+1)

l(1)=alfa(1)*(t-n1-2)+beta(1);

l(2)=alfa(2)*(t-n1-2)+beta(2);

l(3)=alfa(3)*(t-n1-2)+beta(3);

l(4)=alfa(4)*(t-n1-2)+beta(4);

eta(1)=et(1)+(t-n1-1)*ni_21;

eta(2)=et(2)+(t-n1-1)*ni_22;

eta(3)=et(3)+(t-n1-1)*ni_23;

eta(4)=et(4)+(t-n1-1)*ni_24;

apom(1)=a*l(1);

apom(2)=a*l(2);

apom(3)=a*l(3);

apom(4)=a*l(4);

rpom=X(t-1)/(eta(1)/apom(1)+eta(2)/apom(2)+eta(3)/apom(3)+eta(4)/apom(4));

X1(t-1)=floor((eta(1)/apom(1))*rpom);

X2(t-1)=floor((eta(2)/apom(2))*rpom);

X3(t-1)=floor((eta(3)/apom(3))*rpom);

X4(t-1)=floor((eta(4)/apom(4))*rpom);

l(1)=l(1)+alfa(1);

l(2)=l(2)+alfa(2);

l(3)=l(3)+alfa(3);

l(4)=l(4)+alfa(4);

apom(1)=a*l(1);

apom(2)=a*l(2);

apom(3)=a*l(3);

apom(4)=a*l(4);

etapo1(t-1)=eta(1);

etapo2(t-1)=eta(2);

etapo3(t-1)=eta(3);

etapo4(t-1)=eta(4);

apo1(t-1)=a*l(1);

apo2(t-1)=a*l(2);

apo3(t-1)=a*l(3);

apo4(t-1)=a*l(4);

for i=1:4

c(i)=2*eta(i)*ro*w/(a*l(i));

d(i)=alfa(i)/(l(i));

end

O(1)=0;

O(2)=0;

O(3)=0;

for i=1:X1(t-1)

pom1=rand();

if (pom1>p20) && (pom1<=p20+p21)

O(1)=O(1)+1;

end

if (pom1>p20+p21)

O(1)=O(1)+2;

end

end

for i=1:X2(t-1)

pom1=rand();

if (pom1>q20) && (pom1<=q20+q21)

O(2)=O(2)+1;

end

if (pom1>q20+q21)

O(2)=O(2)+2;

end

end

for i=1:X3(t-1)

pom1=rand();

if (pom1>r20) && (pom1<=r20+r21)

O(3)=O(3)+1;

end

if (pom1>r20+r21)

O(3)=O(3)+2;

end

end

rpom=X(t-1)/(eta_1/apom(1)+eta_2/apom(2)+eta_3/apom(3)+eta_4/apom(4));

v=rpom*delta2*(2-delta2)/(2*ro*w);

u=1/(1+c(1)+c(2)+c(3)+c(4))*(d(1)*X1(t-1)+d(2)*X2(t-1)+d(3)*X3(t-1)+d(4)*X4(t-1)+O(1)-X1(t-1)+O(2)-X2(t-1)+O(3)-X3(t-1)-v);

xpom=c(1)*u-(O(1)-X1(t-1))-d(1)*X1(t-1);

ypom=u*(1+c(3)+c(4))-d(3)*X3(t-1)-d(4)*X4(t-1)-(O(3)-X3(t-1))+v;

zpom=u*(1+c(4))-d(4)*X4(t-1)+v;

un=floor(u);

xn=floor(xpom);

yn=floor(ypom);

zn=floor(zpom);

X1(t)=O(1)+xn;

X2(t)=O(2)-xn-yn;

X3(t)=O(3)-zn+yn;

X4(t)=X4(t-1)+zn-floor(u+v);

R(t)=sqrt((X1(t)*a*l(1))/(2*pi*eta(1)));

X(t)=X1(t)+X2(t)+X3(t)+X4(t);

X21(t-1)=xn;

X23(t-1)=yn;

X34(t-1)=zn;

XEbes(t-1)=floor(u+v);

X1pom(t)=X1(t);

X2pom(t)=X2(t);

X3pom(t)=X3(t);

X4pom(t)=X4(t);

end

beta(1)=n2*alfa_21+beta(1);

beta(2)=n2*alfa_22+beta(2);

beta(3)=n2*alfa_23+beta(3);

beta(4)=n2*alfa_24+beta(4);

alfa(1)=alfa_31;

alfa(2)=alfa_32;

alfa(3)=alfa_33;

alfa(4)=alfa_34;

et(1)=n2*ni_21+et(1);

et(2)=n2*ni_22+et(2);

et(3)=n2*ni_23+et(3);

et(4)=n2*ni_24+et(4);

for t=(2+n1+n2):(n1+n2+n3+1)

l(1)=alfa(1)*(t-n1-n2-2)+beta(1);

l(2)=alfa(2)*(t-n1-n2-2)+beta(2);

l(3)=alfa(3)*(t-n1-n2-2)+beta(3);

l(4)=alfa(4)*(t-n1-n2-2)+beta(4);

eta(1)=et(1)+(t-n1-n2-1)*ni_31;

eta(2)=et(2)+(t-n1-n2-1)*ni_32;

eta(3)=et(3)+(t-n1-n2-1)*ni_33;

eta(4)=et(4)+(t-n1-n2-1)*ni_34;

apom(1)=a*l(1);

apom(2)=a*l(2);

apom(3)=a*l(3);

apom(4)=a*l(4);

rpom=X(t-1)/(eta(1)/apom(1)+eta(2)/apom(2)+eta(3)/apom(3)+eta(4)/apom(4));

X1(t-1)=floor((eta(1)/apom(1))*rpom);

X2(t-1)=floor((eta(2)/apom(2))*rpom);

X3(t-1)=floor((eta(3)/apom(3))*rpom);

X4(t-1)=floor((eta(4)/apom(4))*rpom);

l(1)=l(1)+alfa(1);

l(2)=l(2)+alfa(2);

l(3)=l(3)+alfa(3);

l(4)=l(4)+alfa(4);

apom(1)=a*l(1);

apom(2)=a*l(2);

apom(3)=a*l(3);

apom(4)=a*l(4);

etapo1(t-1)=eta(1);

etapo2(t-1)=eta(2);

etapo3(t-1)=eta(3);

etapo4(t-1)=eta(4);

apo1(t-1)=a*l(1);

apo2(t-1)=a*l(2);

apo3(t-1)=a*l(3);

apo4(t-1)=a*l(4);

for i=1:4

c(i)=2*eta(i)*ro*w/(a*l(i));

d(i)=alfa(i)/(l(i));

end

O(1)=0;

O(2)=0;

O(3)=0;

for i=1:X1(t-1)

pom1=rand();

if (pom1>p30) && (pom1<=p30+p31)

O(1)=O(1)+1;

end

if (pom1>p30+p31)

O(1)=O(1)+2;

end

end

for i=1:X2(t-1)

pom1=rand();

if (pom1>q30) && (pom1<=q30+q31)

O(2)=O(2)+1;

end

if (pom1>q30+q31)

O(2)=O(2)+2;

end

end

for i=1:X3(t-1)

pom1=rand();

if (pom1>r30) && (pom1<=r30+r31)

O(3)=O(3)+1;

end

if (pom1>r30+r31)

O(3)=O(3)+2;

end

end

rpom=X(t-1)/(eta_1/apom(1)+eta_2/apom(2)+eta_3/apom(3)+eta_4/apom(4));

v=rpom*delta3*(2-delta3)/(2*ro*w);

u=1/(1+c(1)+c(2)+c(3)+c(4))*(d(1)*X1(t-1)+d(2)*X2(t-1)+d(3)*X3(t-1)+d(4)*X4(t-1)+O(1)-X1(t-1)+O(2)-X2(t-1)+O(3)-X3(t-1)-v);

xpom=c(1)*u-(O(1)-X1(t-1))-d(1)*X1(t-1);

ypom=u*(1+c(3)+c(4))-d(3)*X3(t-1)-d(4)*X4(t-1)-(O(3)-X3(t-1))+v;

zpom=u*(1+c(4))-d(4)*X4(t-1)+v;

un=floor(u);

xn=floor(xpom);

yn=floor(ypom);

zn=floor(zpom);

X1(t)=O(1)+xn;

X2(t)=O(2)-xn-yn;

X3(t)=O(3)-zn+yn;

X4(t)=X4(t-1)+zn-floor(u+v);

R(t)=sqrt((X1(t)*a*l(1))/(2*pi*eta(1)));

X(t)=X1(t)+X2(t)+X3(t)+X4(t);

X21(t-1)=xn;

X23(t-1)=yn;

X34(t-1)=zn;

XEbes(t-1)=floor(u+v);

X1pom(t)=X1(t);

X2pom(t)=X2(t);

X3pom(t)=X3(t);

X4pom(t)=X4(t);

end

beta(1)=n3*alfa_31+beta(1);

beta(2)=n3*alfa_32+beta(2);

beta(3)=n3*alfa_33+beta(3);

beta(4)=n3*alfa_34+beta(4);

alfa(1)=alfa_41;

alfa(2)=alfa_42;

alfa(3)=alfa_43;

alfa(4)=alfa_44;

et(1)=n3*ni_31+et(1);

et(2)=n3*ni_32+et(2);

et(3)=n3*ni_33+et(3);

et(4)=n3*ni_34+et(4);

for t=(2+n1+n2+n3):(n1+n2+n3+n4+1)

l(1)=alfa(1)*(t-n1-n2-n3-2)+beta(1);

l(2)=alfa(2)*(t-n1-n2-n3-2)+beta(2);

l(3)=alfa(3)*(t-n1-n2-n3-2)+beta(3);

l(4)=alfa(4)*(t-n1-n2-n3-2)+beta(4);

eta(1)=et(1)+(t-n1-n2-n3-1)*ni_41;

eta(2)=et(2)+(t-n1-n2-n3-1)*ni_42;

eta(3)=et(3)+(t-n1-n2-n3-1)*ni_43;

eta(4)=et(4)+(t-n1-n2-n3-1)*ni_44;

apom(1)=a*l(1);

apom(2)=a*l(2);

apom(3)=a*l(3);

apom(4)=a*l(4);

rpom=X(t-1)/(eta(1)/apom(1)+eta(2)/apom(2)+eta(3)/apom(3)+eta(4)/apom(4));

X1(t-1)=floor((eta(1)/apom(1))*rpom);

X2(t-1)=floor((eta(2)/apom(2))*rpom);

X3(t-1)=floor((eta(3)/apom(3))*rpom);

X4(t-1)=floor((eta(4)/apom(4))*rpom);

l(1)=l(1)+alfa(1);

l(2)=l(2)+alfa(2);

l(3)=l(3)+alfa(3);

l(4)=l(4)+alfa(4);

apom(1)=a*l(1);

apom(2)=a*l(2);

apom(3)=a*l(3);

apom(4)=a*l(4);

etapo1(t-1)=eta(1);

etapo2(t-1)=eta(2);

etapo3(t-1)=eta(3);

etapo4(t-1)=eta(4);

apo1(t-1)=a*l(1);

apo2(t-1)=a*l(2);

apo3(t-1)=a*l(3);

apo4(t-1)=a*l(4);

for i=1:4

c(i)=2*eta(i)*ro*w/(a*l(i));

d(i)=alfa(i)/(l(i));

end

O(1)=0;

O(2)=0;

O(3)=0;

for i=1:X1(t-1)

pom1=rand();

if (pom1>p40) && (pom1<=p40+p41)

O(1)=O(1)+1;

end

if (pom1>p40+p41)

O(1)=O(1)+2;

end

end

for i=1:X2(t-1)

pom1=rand();

if (pom1>q40) && (pom1<=q40+q41)

O(2)=O(2)+1;

end

if (pom1>q40+q41)

O(2)=O(2)+2;

end

end

for i=1:X3(t-1)

pom1=rand();

if (pom1>r40) && (pom1<=r40+r41)

O(3)=O(3)+1;

end

if (pom1>r40+r41)

O(3)=O(3)+2;

end

end

rpom=X(t-1)/(eta_1/apom(1)+eta_2/apom(2)+eta_3/apom(3)+eta_4/apom(4));

v=rpom*delta4*(2-delta4)/(2*ro*w);

u=1/(1+c(1)+c(2)+c(3)+c(4))*(d(1)*X1(t-1)+d(2)*X2(t-1)+d(3)*X3(t-1)+d(4)*X4(t-1)+O(1)-X1(t-1)+O(2)-X2(t-1)+O(3)-X3(t-1)-v);

xpom=c(1)*u-(O(1)-X1(t-1))-d(1)*X1(t-1);

ypom=u*(1+c(3)+c(4))-d(3)*X3(t-1)-d(4)*X4(t-1)-(O(3)-X3(t-1))+v;

zpom=u*(1+c(4))-d(4)*X4(t-1)+v;

un=floor(u);

xn=floor(xpom);

yn=floor(ypom);

zn=floor(zpom);

X1(t)=O(1)+xn;

X2(t)=O(2)-xn-yn;

X3(t)=O(3)-zn+yn;

X4(t)=X4(t-1)+zn-floor(u+v);

R(t)=sqrt((X1(t)*a*l(1))/(2*pi*eta(1)));

X(t)=X1(t)+X2(t)+X3(t)+X4(t);

X21(t-1)=xn;

X23(t-1)=yn;

X34(t-1)=zn;

XEbes(t-1)=floor(u+v);

X1pom(t)=X1(t);

X2pom(t)=X2(t);

X3pom(t)=X3(t);

X4pom(t)=X4(t);

end

et(1)=eta_5;

et(2)=eta_6;

et(3)=eta_7;

et(4)=eta_8;

beta(1)=n4*alfa_41+beta(1);

beta(2)=n4*alfa_42+beta(2);

beta(3)=n4*alfa_43+beta(3);

beta(4)=n4*alfa_44+beta(4);

alfa(1)=alfa_51;

alfa(2)=alfa_52;

alfa(3)=alfa_53;

alfa(4)=alfa_54;

for t=(2+n1+n2+n3+n4):(n5+n1+n2+n3+n4+1)

l(1)=alfa(1)*(t-n1-n2-n3-n4-1)+beta(1);

apom(1)=a*(alfa(1)*(t-n1-n2-n3-n4-2)+beta(1));

l(2)=alfa(2)*(t-n1-n2-n3-n4-1)+beta(2);

apom(2)=a*(alfa(2)*(t-n1-n2-n3-n4-2)+beta(2));

l(3)=alfa(3)*(t-n1-n2-n3-n4-1)+beta(3);

apom(3)=a*(alfa(3)*(t-n1-n2-n3-n4-2)+beta(3));

l(4)=alfa(4)*(t-n1-n2-n3-n4-1)+beta(4);

apom(4)=a*(alfa(4)*(t-n1-n2-n3-n4-2)+beta(4));

eta(1)=et(1)+(t-n1-n2-n3-n4-1)*ni_51;

eta(2)=et(2)+(t-n1-n2-n3-n4-1)*ni_52;

eta(3)=et(3)+(t-n1-n2-n3-n4-1)*ni_53;

eta(4)=et(4)+(t-n1-n2-n3-n4-1)*ni_54;

etapo1(t-1)=eta(1);

etapo2(t-1)=eta(2);

etapo3(t-1)=eta(3);

etapo4(t-1)=eta(4);

apo1(t-1)=a*l(1);

apo2(t-1)=a*l(2);

apo3(t-1)=a*l(3);

apo4(t-1)=a*l(4);

for i=1:4

c(i)=2*eta(i)*ro*w/(a*l(i));

d(i)=alfa(i)/(l(i));

end

O(1)=0;

O(2)=0;

O(3)=0;

for i=1:X1(t-1)

pom1=rand();

if (pom1>p50) && (pom1<=p50+p51)

O(1)=O(1)+1;

end

if (pom1>p50+p51)

O(1)=O(1)+2;

end

end

for i=1:X2(t-1)

pom1=rand();

if (pom1>q50) && (pom1<=q50+q51)

O(2)=O(2)+1;

end

if (pom1>q50+q51)

O(2)=O(2)+2;

end

end

for i=1:X3(t-1)

pom1=rand();

if (pom1>r50) && (pom1<=r50+r51)

O(3)=O(3)+1;

end

if (pom1>r50+r51)

O(3)=O(3)+2;

end

end

rpom=X(t-1)/(eta_5/apom(1)+eta_6/apom(2)+eta_7/apom(3)+eta_8/apom(4));

v=rpom*delta5*(2-delta5)/(2*ro*w);

u=1/(1+c(1)+c(2)+c(3)+c(4))*(d(1)*X1(t-1)+d(2)*X2(t-1)+d(3)*X3(t-1)+d(4)*X4(t-1)+O(1)-X1(t-1)+O(2)-X2(t-1)+O(3)-X3(t-1)-v);

xpom=c(1)*u-(O(1)-X1(t-1))-d(1)*X1(t-1);

ypom=u*(1+c(3)+c(4))-d(3)*X3(t-1)-d(4)*X4(t-1)-(O(3)-X3(t-1))+v;

zpom=u*(1+c(4))-d(4)*X4(t-1)+v;

un=floor(u);

xn=floor(xpom);

yn=floor(ypom);

zn=floor(zpom);

X1(t)=O(1)+xn;

X2(t)=O(2)-xn-yn;

X3(t)=O(3)-zn+yn;

X4(t)=X4(t-1)+zn-floor(u+v);

R(t)=sqrt((X1(t)*a*l(1))/(2*pi*eta(1)));

X(t)=X1(t)+X2(t)+X3(t)+X4(t);

X21(t-1)=xn;

X23(t-1)=yn;

X34(t-1)=zn;

XEbes(t-1)=floor(u+v);

end

beta(1)=n5*alfa_51+beta(1);

beta(2)=n5*alfa_52+beta(2);

beta(3)=n5*alfa_53+beta(3);

beta(4)=n5*alfa_54+beta(4);

alfa(1)=alfa_61;

alfa(2)=alfa_62;

alfa(3)=alfa_63;

alfa(4)=alfa_64;

et(1)=n5*ni_51+et(1);

et(2)=n5*ni_52+et(2);

et(3)=n5*ni_53+et(3);

et(4)=n5*ni_54+et(4);

ro=10.7;

for t=(2+n1+n2+n3+n4+n5):(n1+n2+n3+n4+n5+n6+1)

l(1)=alfa(1)*(t-n5-n1-n2-n3-n4-1)+beta(1);

apom(1)=a*(alfa(1)*(t-n5-n1-n2-n3-n4-2)+beta(1));

l(2)=alfa(2)*(t-n5-n1-n2-n3-n4-1)+beta(2);

apom(2)=a*(alfa(2)*(t-n5-n1-n2-n3-n4-2)+beta(2));

l(3)=alfa(3)*(t-n5-n1-n2-n3-n4-1)+beta(3);

apom(3)=a*(alfa(3)*(t-n5-n1-n2-n3-n4-2)+beta(3));

l(4)=alfa(4)*(t-n5-n1-n2-n3-n4-1)+beta(4);

apom(4)=a*(alfa(4)*(t-n5-n1-n2-n3-n4-2)+beta(4));

eta(1)=et(1)+(t-n1-n2-n3-n4-n5-1)*ni_61;

eta(2)=et(2)+(t-n1-n2-n3-n4-n5-1)*ni_62;

eta(3)=et(3)+(t-n1-n2-n3-n4-n5-1)*ni_63;

eta(4)=et(4)+(t-n1-n2-n3-n4-n5-1)*ni_64;

etapo1(t-1)=eta(1);

etapo2(t-1)=eta(2);

etapo3(t-1)=eta(3);

etapo4(t-1)=eta(4);

apo1(t-1)=a*l(1);

apo2(t-1)=a*l(2);

apo3(t-1)=a*l(3);

apo4(t-1)=a*l(4);

for i=1:4

c(i)=2*eta(i)*ro*w/(a*l(i));

d(i)=alfa(i)/(l(i));

end

O(1)=0;

O(2)=0;

O(3)=0;

for i=1:X1(t-1)

pom1=rand();

if (pom1>p60) && (pom1<=p60+p61)

O(1)=O(1)+1;

end

if (pom1>p60+p61)

O(1)=O(1)+2;

end

end

for i=1:X2(t-1)

pom1=rand();

if (pom1>q60) && (pom1<=q60+q61)

O(2)=O(2)+1;

end

if (pom1>q60+q61)

O(2)=O(2)+2;

end

end

for i=1:X3(t-1)

pom1=rand();

if (pom1>r60) && (pom1<=r60+r61)

O(3)=O(3)+1;

end

if (pom1>r60+r61)

O(3)=O(3)+2;

end

end

rpom=X(t-1)/(eta_5/apom(1)+eta_6/apom(2)+eta_7/apom(3)+eta_8/apom(4));

v=rpom*delta6*(2-delta6)/(2*ro*w);

u=1/(1+c(1)+c(2)+c(3)+c(4))*(d(1)*X1(t-1)+d(2)*X2(t-1)+d(3)*X3(t-1)+d(4)*X4(t-1)+O(1)-X1(t-1)+O(2)-X2(t-1)+O(3)-X3(t-1)-v);

xpom=c(1)*u-(O(1)-X1(t-1))-d(1)*X1(t-1);

ypom=u*(1+c(3)+c(4))-d(3)*X3(t-1)-d(4)*X4(t-1)-(O(3)-X3(t-1))+v;

zpom=u*(1+c(4))-d(4)*X4(t-1)+v;

un=floor(u);

xn=floor(xpom);

yn=floor(ypom);

zn=floor(zpom);

X1(t)=O(1)+xn;

X2(t)=O(2)-xn-yn;

X3(t)=O(3)-zn+yn;

X4(t)=X4(t-1)+zn-floor(u+v);

R(t)=sqrt((X1(t)*a*l(1))/(2*pi*eta(1)));

X(t)=X1(t)+X2(t)+X3(t)+X4(t);

X21(t-1)=xn;

X23(t-1)=yn;

X34(t-1)=zn;

XEbes(t-1)=floor(u+v);

end

beta(1)=n6*alfa_61+beta(1);

beta(2)=n6*alfa_62+beta(2);

beta(3)=n6*alfa_63+beta(3);

beta(4)=n6*alfa_64+beta(4);

alfa(1)=alfa_71;

alfa(2)=alfa_72;

alfa(3)=alfa_73;

alfa(4)=alfa_74;

et(1)=n6*ni_61+et(1);

et(2)=n6*ni_62+et(2);

et(3)=n6*ni_63+et(3);

et(4)=n6*ni_64+et(4);

ro=11.5;

for t=(2+n1+n2+n3+n4+n5+n6):(n1+n2+n3+n4+n5+n6+n7+1)

l(1)=alfa(1)*(t-n5-n6-n1-n2-n3-n4-1)+beta(1);

apom(1)=a*(alfa(1)*(t-n5-n6-n1-n2-n3-n4-2)+beta(1));

l(2)=alfa(2)*(t-n5-n6-n1-n2-n3-n4-1)+beta(2);

apom(2)=a*(alfa(2)*(t-n5-n6-n1-n2-n3-n4-2)+beta(2));

l(3)=alfa(3)*(t-n5-n6-n1-n2-n3-n4-1)+beta(3);

apom(3)=a*(alfa(3)*(t-n5-n6-n1-n2-n3-n4-2)+beta(3));

l(4)=alfa(4)*(t-n5-n6-n1-n2-n3-n4-1)+beta(4);

apom(4)=a*(alfa(4)*(t-n5-n1-n2-n3-n4-n6-2)+beta(4));

eta(1)=et(1)+(t-n1-n2-n3-n4-n5-n6-1)*ni_71;

eta(2)=et(2)+(t-n1-n2-n3-n4-n5-n6-1)*ni_72;

eta(3)=et(3)+(t-n1-n2-n3-n4-n5-n6-1)*ni_73;

eta(4)=et(4)+(t-n1-n2-n3-n4-n5-n6-1)*ni_74;

etapo1(t-1)=eta(1);

etapo2(t-1)=eta(2);

etapo3(t-1)=eta(3);

etapo4(t-1)=eta(4);

apo1(t-1)=a*l(1);

apo2(t-1)=a*l(2);

apo3(t-1)=a*l(3);

apo4(t-1)=a*l(4);

for i=1:4

c(i)=2*eta(i)*ro*w/(a*l(i));

d(i)=alfa(i)/(l(i));

end

O(1)=0;

O(2)=0;

O(3)=0;

for i=1:X1(t-1)

pom1=rand();

if (pom1>p70) && (pom1<=p70+p71)

O(1)=O(1)+1;

end

if (pom1>p70+p71)

O(1)=O(1)+2;

end

end

for i=1:X2(t-1)

pom1=rand();

if (pom1>q70) && (pom1<=q70+q71)

O(2)=O(2)+1;

end

if (pom1>q70+q71)

O(2)=O(2)+2;

end

end

for i=1:X3(t-1)

pom1=rand();

if (pom1>r70) && (pom1<=r70+r71)

O(3)=O(3)+1;

end

if (pom1>r70+r71)

O(3)=O(3)+2;

end

end

rpom=X(t-1)/(eta_5/apom(1)+eta_6/apom(2)+eta_7/apom(3)+eta_8/apom(4));

v=rpom*delta7*(2-delta7)/(2*ro*w);

u=1/(1+c(1)+c(2)+c(3)+c(4))*(d(1)*X1(t-1)+d(2)*X2(t-1)+d(3)*X3(t-1)+d(4)*X4(t-1)+O(1)-X1(t-1)+O(2)-X2(t-1)+O(3)-X3(t-1)-v);

xpom=c(1)*u-(O(1)-X1(t-1))-d(1)*X1(t-1);

ypom=u*(1+c(3)+c(4))-d(3)*X3(t-1)-d(4)*X4(t-1)-(O(3)-X3(t-1))+v;

zpom=u*(1+c(4))-d(4)*X4(t-1)+v;

un=floor(u);

xn=floor(xpom);

yn=floor(ypom);

zn=floor(zpom);

X1(t)=O(1)+xn;

X2(t)=O(2)-xn-yn;

X3(t)=O(3)-zn+yn;

X4(t)=X4(t-1)+zn-floor(u+v);

R(t)=sqrt((X1(t)*a*l(1))/(2*pi*eta(1)));

X(t)=X1(t)+X2(t)+X3(t)+X4(t);

X21(t-1)=xn;

X23(t-1)=yn;

X34(t-1)=zn;

XEbes(t-1)=floor(u+v);

end

beta(1)=n7*alfa_71+beta(1);

beta(2)=n7*alfa_72+beta(2);

beta(3)=n7*alfa_73+beta(3);

beta(4)=n7*alfa_74+beta(4);

alfa(1)=alfa_81;

alfa(2)=alfa_82;

alfa(3)=alfa_83;

alfa(4)=alfa_84;

et(1)=n7*ni_71+et(1);

et(2)=n7*ni_72+et(2);

et(3)=n7*ni_73+et(3);

et(4)=n7*ni_74+et(4);

ro=12.5;

for t=(2+n1+n2+n3+n4+n5+n6+n7):(n1+n2+n3+n4+n5+n6+n7+n8+1)

l(1)=alfa(1)*(t-n5-n6-n7-n1-n2-n3-n4-1)+beta(1);

apom(1)=a*(alfa(1)*(t-n5-n6-n7-n1-n2-n3-n4-2)+beta(1));

l(2)=alfa(2)*(t-n5-n6-n7-n1-n2-n3-n4-1)+beta(2);

apom(2)=a*(alfa(2)*(t-n5-n6-n7-n1-n2-n3-n4-2)+beta(2));

l(3)=alfa(3)*(t-n5-n6-n7-n1-n2-n3-n4-1)+beta(3);

apom(3)=a*(alfa(3)*(t-n5-n6-n7-n1-n2-n3-n4-2)+beta(3));

l(4)=alfa(4)*(t-n5-n6-n7-n1-n2-n3-n4-1)+beta(4);

apom(4)=a*(alfa(4)*(t-n5-n6-n7-n1-n2-n3-n4-2)+beta(4));

eta(1)=et(1)+(t-n1-n2-n3-n4-n5-n6-n7-1)*ni_81;

eta(2)=et(2)+(t-n1-n2-n3-n4-n5-n6-n7-1)*ni_82;

eta(3)=et(3)+(t-n1-n2-n3-n4-n5-n6-n7-1)*ni_83;

eta(4)=et(4)+(t-n1-n2-n3-n4-n5-n6-n7-1)*ni_84;

etapo1(t-1)=eta(1);

etapo2(t-1)=eta(2);

etapo3(t-1)=eta(3);

etapo4(t-1)=eta(4);

apo1(t-1)=a*l(1);

apo2(t-1)=a*l(2);

apo3(t-1)=a*l(3);

apo4(t-1)=a*l(4);

for i=1:4

c(i)=2*eta(i)*ro*w/(a*l(i));

d(i)=alfa(i)/(l(i));

end

O(1)=0;

O(2)=0;

O(3)=0;

for i=1:X1(t-1)

pom1=rand();

if (pom1>p80) && (pom1<=p80+p81)

O(1)=O(1)+1;

end

if (pom1>p80+p81)

O(1)=O(1)+2;

end

end

for i=1:X2(t-1)

pom1=rand();

if (pom1>q80) && (pom1<=q80+q81)

O(2)=O(2)+1;

end

if (pom1>q80+q81)

O(2)=O(2)+2;

end

end

for i=1:X3(t-1)

pom1=rand();

if (pom1>r80) && (pom1<=r80+r81)

O(3)=O(3)+1;

end

if (pom1>r80+r81)

O(3)=O(3)+2;

end

end

rpom=X(t-1)/(eta_5/apom(1)+eta_6/apom(2)+eta_7/apom(3)+eta_8/apom(4));

v=rpom*delta8*(2-delta8)/(2*ro*w);

u=1/(1+c(1)+c(2)+c(3)+c(4))*(d(1)*X1(t-1)+d(2)*X2(t-1)+d(3)*X3(t-1)+d(4)*X4(t-1)+O(1)-X1(t-1)+O(2)-X2(t-1)+O(3)-X3(t-1)-v);

xpom=c(1)*u-(O(1)-X1(t-1))-d(1)*X1(t-1);

ypom=u*(1+c(3)+c(4))-d(3)*X3(t-1)-d(4)*X4(t-1)-(O(3)-X3(t-1))+v;

zpom=u*(1+c(4))-d(4)*X4(t-1)+v;

un=floor(u);

xn=floor(xpom);

yn=floor(ypom);

zn=floor(zpom);

X1(t)=O(1)+xn;

X2(t)=O(2)-xn-yn;

X3(t)=O(3)-zn+yn;

X4(t)=X4(t-1)+zn-floor(u+v);

R(t)=sqrt((X1(t)*a*l(1))/(2*pi*eta(1)));

X(t)=X1(t)+X2(t)+X3(t)+X4(t);

X21(t-1)=xn;

X23(t-1)=yn;

X34(t-1)=zn;

XEbes(t-1)=floor(u+v);

end

n=n1+n2+n3+n4+n5+n6+n7+n8;

npom=n1+n2+n3+n4;

var(1,:)=X;

var(2,:)=R;

var(3,1:npom)=X1pom(1:npom);

var(3,(npom+1):(n+1))=X1((npom+1):(n+1));

var(4,1:npom)=X2pom(1:npom);

var(4,(npom+1):(n+1))=X2((npom+1):(n+1));

var(5,1:npom)=X3pom(1:npom);

var(5,(npom+1):(n+1))=X3((npom+1):(n+1));

var(6,1:npom)=X4pom(1:npom);

var(6,(npom+1):(n+1))=X4((npom+1):(n+1));

var(7,1:n)=X21;

var(8,1:n)=X23;

var(9,1:n)=X34;

var(10,1:n)=XEbes;

var(11,(npom+1):(npom+n5))=r52;

var(11,(npom+n5+1):(npom+n5+n6))=r62;

var(11,(npom+n5+n6+1):(npom+n5+n6+n7))=r72;

var(11,(npom+n5+n6+n7+1):n)=r82;

var(12,1:n)=apo1(1:n);

var(13,1:n)=apo2(1:n);

var(14,1:n)=apo3(1:n);

var(15,1:n)=apo4(1:n);

var(16,1:n)=etapo1(1:n);

var(17,1:n)=etapo2(1:n);

var(18,1:n)=etapo3(1:n);

var(19,1:n)=etapo4(1:n);

end
